# Tumor suppressor NDRG2 inhibits glycolysis and glutaminolysis in colorectal cancer cells by repressing c-Myc expression

**DOI:** 10.18632/oncotarget.4544

**Published:** 2015-07-20

**Authors:** Xinyuan Xu, Jianying Li, Xiang Sun, Yan Guo, Dake Chu, Li Wei, Xia Li, Guodong Yang, Xinping Liu, Libo Yao, Jian Zhang, Lan Shen

**Affiliations:** ^1^ The State Key Laboratory of Cancer Biology, Department of Biochemistry and Molecular Biology, The Fourth Military Medical University, Xi'an, Shaanxi, China; ^2^ Department of Prosthodontics, School of Stomatology, The Fourth Military Medical University, Xi'an, Shaanxi, China; ^3^ The State Key Laboratory of Cancer Biology, Department of Gastrointestinal Surgery, Xijing Hospital of Digestive Diseases, The Fourth Military Medical University, Xi'an, Shaanxi, China; ^4^ Department of Obstetrics and Gynecology, Xijing Hospital, The Fourth Military Medical University, Xi'an, Shaanxi, China

**Keywords:** NDRG2, tumor metabolism, aerobic glycolysis, glutaminolysis, colorectal cancer

## Abstract

Cancer cells use glucose and glutamine as the major sources of energy and precursor intermediates, and enhanced glycolysis and glutamimolysis are the major hallmarks of metabolic reprogramming in cancer. Oncogene activation and tumor suppressor gene inactivation alter multiple intracellular signaling pathways that affect glycolysis and glutaminolysis. *N-Myc downstream regulated gene 2* (*NDRG2*) is a tumor suppressor gene inhibiting cancer growth, metastasis and invasion. However, the role and molecular mechanism of NDRG2 in cancer metabolism remains unclear. In this study, we discovered the role of the tumor suppressor gene NDRG2 in aerobic glycolysis and glutaminolysis of cancer cells. NDRG2 inhibited glucose consumption and lactate production, glutamine consumption and glutamate production in colorectal cancer cells. Analysis of glucose transporters and the catalytic enzymes involved in glycolysis revealed that glucose transporter 1 (GLUT1), hexokinase 2 (HK2), pyruvate kinase M2 isoform (PKM2) and lactate dehydrogenase A (LDHA) was significantly suppressed by NDRG2. Analysis of glutamine transporter and the catalytic enzymes involved in glutaminolysis revealed that glutamine transporter ASC amino-acid transporter 2 (ASCT2) and glutaminase 1 (GLS1) was also significantly suppressed by NDRG2. Transcription factor c-Myc mediated inhibition of glycolysis and glutaminolysis by NDRG2. More importantly, NDRG2 inhibited the expression of c-Myc by suppressing the expression of β-catenin, which can transcriptionally activate *C-MYC* gene in nucleus. In addition, the growth and proliferation of colorectal cancer cells were suppressed significantly by NDRG2 through inhibition of glycolysis and glutaminolysis. Taken together, these findings indicate that NDRG2 functions as an essential regulator in glycolysis and glutaminolysis via repression of c-Myc, and acts as a suppressor of carcinogenesis through coordinately targeting glucose and glutamine transporter, multiple catalytic enzymes involved in glycolysis and glutaminolysis, which fuels the bioenergy and biomaterials needed for cancer proliferation and progress.

## INTRODUCTION

Malignant growth and proliferation of cancer cells consume large quantities of bioenergy and biomaterials, which enhance the carbon flux through glycolysis and glutaminolysis to fulfill energetic and biosynthetic demands of cancer cells [1, 2]. During the development of cancer, oncogene activation and tumor suppressor gene inactivation cause alterations to multiple intracellular signaling molecules that affect tumor cell glycolytic and glutaminolytic flux [3–6]. Thus, improved understanding of the molecular mechanism involved in tumor metabolic reprogramming, especially in active glycolysis and glutaminolysis, may assist in the discovery of new molecular diagnostic methods and targeted therapeutic strategies for cancer.

Aerobic glycolysis is often accompanied by increased glucose uptake in different types of tumors, with this phenotype serving as the basis for clinical detection of tumors by [18-F]-fluorodeoxyglucose positron emission tomography (FDG-PET). The significant enhancement in tumor [18-F]-FDG uptake could be explained at least in part by increased membrane glucose transporters expression [7, 8]. The expression and activation of glucose transporters are always regulated by several oncogenes and tumor suppressor genes including c-Myc, hypoxia inducible factor-1 (HIF-1), and p53 in cancer cells [9]. Therefore, cell metabolism is shifted toward glycolysis attributes partly to the increased expression of glucose transporters. Currently, increased aerobic glycolysis in cancer cell is also believed to be due to the changes of catalytic enzymes involved in glycolysis, which are regulated by oncogenes and tumor suppressor genes. Firstly, hexokinase 2 (HK2), the first rate-limiting enzyme in the glycolytic pathway, is transcriptionally activated by the oncogenic transcriptional factor Myc [10, 11]. Secondly, the M2 isoform of pyruvate kinase (PKM2), the last rate-limiting enzyme in the glycolytic pathway, is up-regulated in colorectal cancer cells and plays an important role in the regulation of cancer metabolism [12, 13]. PKM2 is regulated by multiple oncogenic transcriptional factors, such as c-Myc, hypoxia-inducible factor 1 (HIF-1), specificity protein (SP) 1, and SP2. PKM2 is also regulated by tumor suppressors tuberous sclerosis complex (TSC1/2) and phosphatase and tensin homolog (PTEN) [13]. Finally, lactate dehydrogenase A (LDHA), which can catalyze pyruvate into lactate, is also directly transactivated by Myc and HIF-1 [6, 14]. Consequently, these metabolic enzymes are important for the dynamic regulation of enhanced glycolytic flux in cancer cells.

Glutaminolysis is the process of glutamine catabolism, which is catalyzed by glutaminase (GLS) and glutamate dehydrogenase (GDH). Glutamine is the most important amino acid in cancer cell metabolism and proliferation, glutamine deprivation induces apoptosis and inhibits growth of neuroblastoma cells [15]. ASC amino-acid transporter 2 (ASCT2) is a major glutamine transporter in cancer cells [16], and activation of ASCT2 captures large amounts of glutamine to support the malignant growth of cancer cells [17]. Therefore, ASCT2-mediated glutamine transport is a potential therapeutic target through inhibiting cancer cell growth and proliferation [18]. Glutaminase 1 (GLS1) is the first rate-limiting enzyme in glutaminolysis which is regulated by transcription factor c-Myc, while GDH is regulated by GTP and leucine [19–22]. In malignant proliferating cancer cells, active glutamine transport and enzymatic activities lead to glutaminolysis addiction.

*N-Myc downstream-regulated gene 2* (*NDRG2*), a member of the *NDRG* family, was firstly discovered in our laboratory by subtractive hybridization [23]. Our previous research indicated that NDRG2 was expressed widely in normal tissues [24], and decreased in colon tumor and other types of tumor tissues [25–30]. Moreover, NDRG2 also inhibited the growth, proliferation and invasion of colon tumor cells and other types of tumor cells [23, 31–35]. Therefore, NDRG2 is classified as the tumor suppressor gene [33, 34, 36]. Besides malignant growth and invasion, metabolic abnormality is currently considered as the new malignant phenotype of cancer cells [37]. The regulatory role and molecular mechanism of NDRG2 in tumor suppression, especially in tumor metabolic reprogramming, remain unclear.

This study aimed to examine whether NDRG2 participates in glycolysis and glutaminolysis in cancer cells, and to clarify the molecular mechanism about NDRG2 regulation of glycolysis and glutaminolysis. Our data demonstrate for the first time that NDRG2 inhibits glycolysis in colorectal cancer cells by inhibiting glucose transporter 1, catalytic enzymes HK2, PKM2, LDHA. Meanwhile, NDRG2 inhibits glutaminolysis in colorectal cancer cells by inhibiting glutamine transporter ASCT2 and glutaminase 1. Oncogenic transcription factor c-Myc mediated inhibition of glycolysis and glutaminolysis by NDRG2. Furthermore, NDRG2 inhibited the expression of c-Myc by suppressing the expression of β-catenin, which can transcriptionally activate *C-MYC* gene in nucleus. Together, the data implicate that *NDRG2* acts as the tumor suppressor gene and participates in the inhibition of glycolysis and glutaminolysis by repression of c-Myc expression in cancer cells. Therefore, NDRG2 might be a potential therapeutic target in targeted cancer therapy.

## RESULTS

### NDRG2 inhibits glycolysis and glutaminolysis in colorectal cancer cells

**Figure 1:**
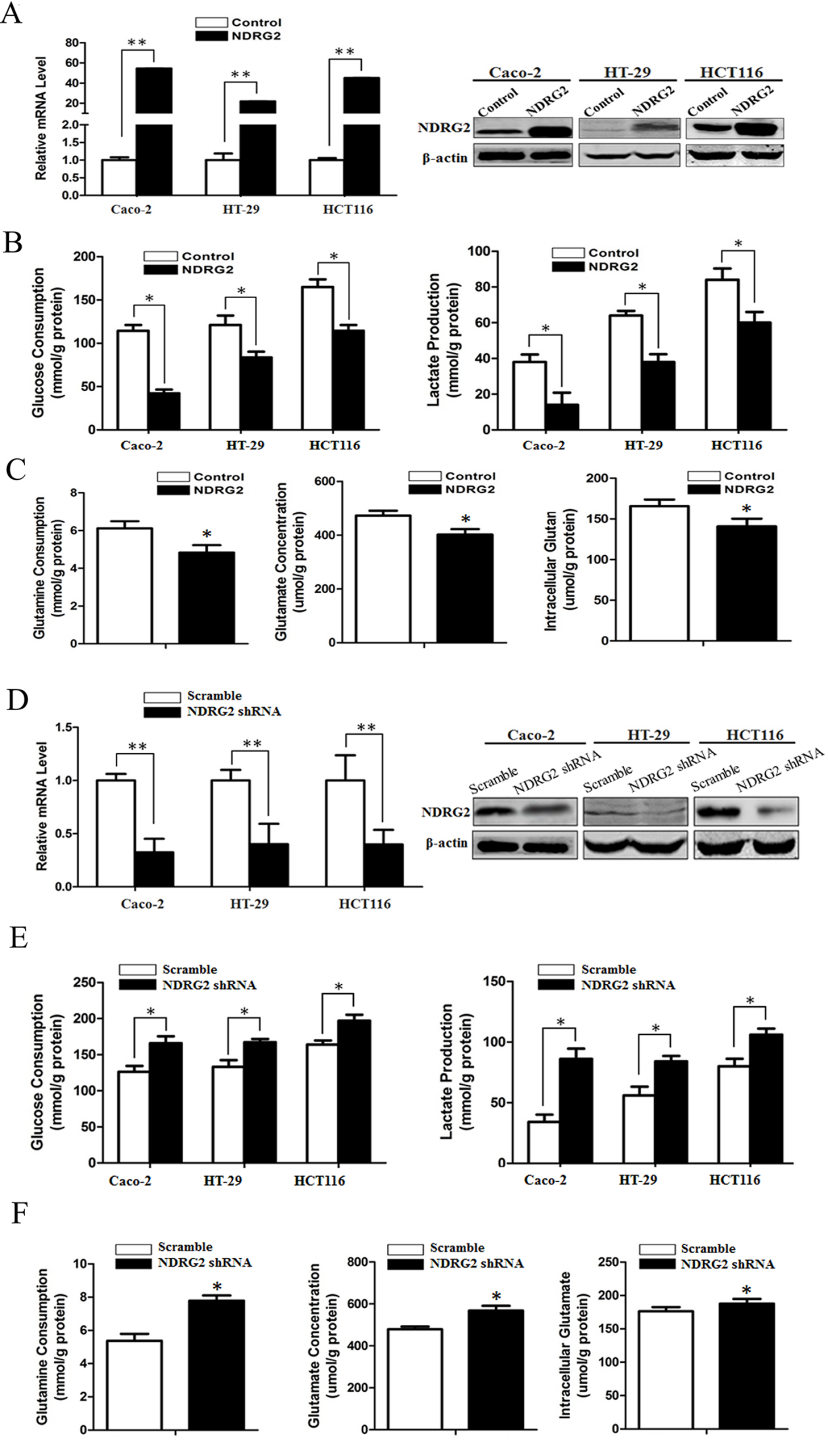
NDRG2 inhibits glycolysis and glutaminolysis in colorectal cancer cells **A.** Quantified NDRG2 mRNA levels and protein levels in Caco-2, HT-29 and HCT116 cells infected with lentivirus containing NDRG2 or mCherry, and β-actin served as an internal control to ensure equal loading. **B.** Glucose consumption and lactate production in NDRG2-overexpressing Caco-2, HT-29 and HCT116 cells were assessed. **C.** Glutamine consumption, glutamate concentration and intracellular glutamate in NDRG2-overexpressing HCT116 cells were assessed. **D.** Quantified NDRG2 mRNA levels and protein levels in Caco-2, HT-29 and HCT116 cells infected with lentivirus containing NDRG2 shRNA or control shRNA, and β-actin served as an internal control to ensure equal loading. **E.** Glucose consumption and lactate production in NDRG2-knockdown Caco-2, HT-29 and HCT116 cells were assessed. **F.** Glutamine consumption, glutamate concentration and intracellular glutamate in NDRG2-knockdown HCT116 cells were assessed. All *P < 0.05; **P < 0.01.

To establish the role of NDRG2 in metabolic reprogramming of colorectal cancer, we used a metabolomics approach to analyze differences among the global metabolic profiles of NDRG2-overexpressing and control HCT116 cells. Metabolites difference and heat map analysis show that glycolytic and glutaminolytic metabolites decreased significantly in NDRG2-overexpressing HCT116 cells (Supplementary Figure S1). Accordingly, overexpression of NDRG2 by lentivirus infection in colorectal cancer cell lines (Figure 1A) inhibited aerobic glycolysis, as indicated by decreased glucose consumption and lactate production in Caco-2, HT-29 and HCT116 cells (Figure 1B), decreased extracellular acidification rate (ECAR) and increased oxygen consumption rate (OCR) in HCT116 cells (Supplementary Figure S2). In addition to NDRG2-mediated inhibition of glycolytic metabolites, overexpression of NDRG2 also inhibited glutaminolysis, as indicated by decreased glutamine consumption, glutamate concentration in the culture medium and intracellular glutamate concentration in HCT116 cells (Figure 1C).

Consistent with the inhibition effect of NDRG2 overexpression on glycolysis and glutaminolysis, knockdown of NDRG2 by lentivirus-mediated shRNA in colorectal cancer cell lines (Figure 1D and Supplementary Figure S3A) facilitated glycolysis and glutaminolysis, as indicated by increased glucose consumption and lactate production in Caco-2, HT-29 and HCT116 cells (Figure 1E and Supplementary Figure S3B), increased glutamine consumption, glutamate concentration in the culture medium and intracellular glutamate concentration in HCT116 cells (Figure 1F and Supplementary Figure S3C). These findings reflected that NDRG2 inhibited glycolytic and glutaminolytic flux in colorectal cancer cells.

### NDRG2 inhibits GLUT1, HK2, PKM2, and LDHA expression in glycolysis of colorectal cancer cells

**Figure 2:**
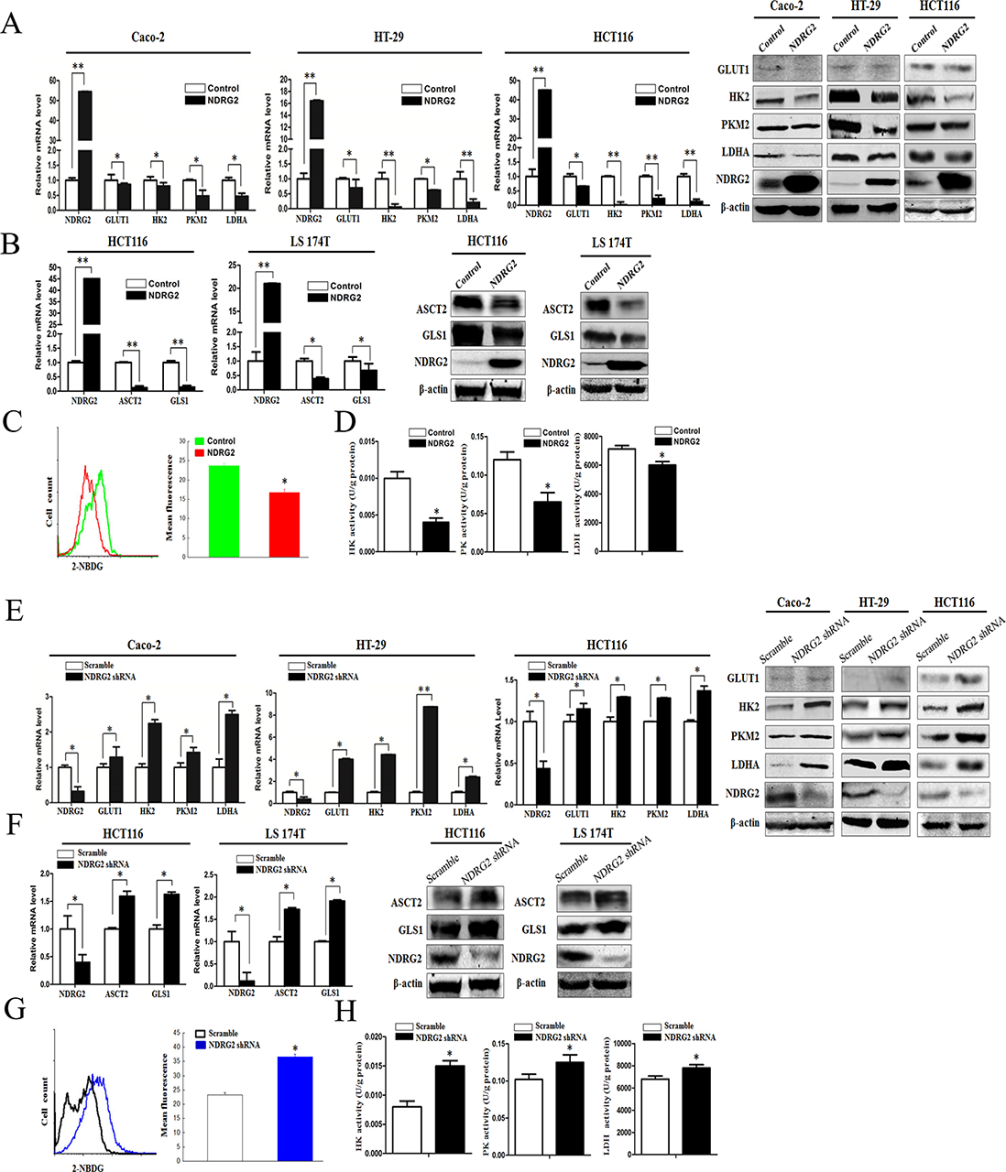
NDRG2 inhibits the expression of glucose and glutamine transporters, glycolytic and glutaminolytic enzymes in colorectal cancer cells **A.** NDRG2, GLUT1, HK2, PKM2, and LDHA mRNA and protein levels in Caco-2, HT-29 and HCT116 cells infected with lentivirus containing NDRG2 or mCherry, and β-actin served as an internal control to ensure equal loading. **B.** NDRG2, ASCT2 and GLS1 mRNA and protein levels in HCT116 and LS 174T cells infected with lentivirus containing NDRG2 or mCherry, and β-actin served as an internal control to ensure equal loading. **C.** Glucose transport activity measured using the fluorescent glucose analog 2-NBDG and **D.** glycolytic enzymes HK, PYK and LDH activity in HT-29 cells infected with lentivirus containing NDRG2 or mCherry. **E.** NDRG2, GLUT1, HK2, PKM2, and LDHA mRNA and protein levels in Caco-2, HT-29 and HCT116 cells infected with lentivirus containing NDRG2 shRNA or control shRNA, and β-actin served as an internal control to ensure equal loading. **F.** NDRG2, ASCT2 and GLS1 mRNA and protein levels in HCT116 and LS 174T cells infected with lentivirus containing NDRG2 shRNA or control shRNA, and β-actin served as an internal control to ensure equal loading. **G.** Glucose transport activity measured using the fluorescent glucose analog 2-NBDG and **H.** glycolytic enzymes HK, PYK and LDH activity in HT-29 cells infected with lentivirus containing NDRG2 shRNA or control shRNA. All *P < 0.05; **P < 0.01.

To identify the underlying target molecules regulated by NDRG2 in tumor aerobic glycolysis, we analyzed the expression of glucose transporters and glycolytic pathway-related enzymes in NDRG2-overexpressing and NDRG2-knockdown Caco-2, HT-29 and HCT116 cells. Interestingly, the expression of glucose transporter 1 (GLUT1), glycolytic pathway-related enzymes HK2, PKM2 and LDHA decreased significantly in NDRG2-overexpressing Caco-2, HT-29 and HCT116 cells (Figure 2A). Next, 2-NBDG uptake revealed that glucose transport activity decreased significantly in NDRG2-overexpressing HT-29 cells (Figure 2C). Meanwhile, enzyme activity analysis revealed that HK, PYK and LDH activity decreased significantly in NDRG2-overexpressing HT-29 cells (Figure 2D). Furthermore, to evaluate the influence of NDRG2 on glucose uptake *in vivo*, nude mice transfected with NDRG2-overexpressing HT-29 cells were prepared for ^18^F-FDG PET scanning. MicroPET scanning demonstrated that ^18^F-FDG accumulation was markedly decreased by NDRG2 overexpression (Supplementary Figure S4).

Consistent with the target molecules inhibited in NDRG2-overexpressing colorectal cancer cells, the expression of GLUT1, glycolytic pathway-related enzymes HK2, PKM2 and LDHA increased significantly in NDRG2-knockdown Caco-2, HT-29 and HCT116 cells (Figure 2E and Supplementary Figure S3D). Furthermore, 2-NBDG uptake and enzyme activity analysis revealed that glucose transport activity, HK, PYK and LDH activity increased significantly in NDRG2-knockdown HT-29 cells (Figure 2G and 2H). These target molecules in glucose transport and catabolism all contributed to NDRG2-induced aerobic glycolysis inhibition in colorectal cancer cells.

### NDRG2 inhibits ASCT2 and GLS1 expression in glutaminolysis of colorectal cancer cells

To identify the underlying target molecules regulated by NDRG2 in tumor glutaminolysis, we analyzed the expression of glutamine transporters and glutaminolytic pathway-related enzymes in NDRG2-overexpressing and NDRG2- knockdown HCT116 cells. Interestingly, the expression of glutamine transporter ASCT2 and glutaminase 1 (GLS1) decreased significantly in NDRG2-overexpressing HCT116, LS 174T and LoVo cells (Figure 2B and Supplementary Figure S5A). Consistent with the target molecules inhibited in NDRG2-overexpressing cells, the expression of glutamine transporter ASCT2 and GLS1 increased significantly in NDRG2-knockdown HCT116, LS 174T and LoVo cells (Figure 2F and Supplementary Figure S5B). These results indicated that ASCT2 and GLS1 both contributed to NDRG2-induced glutaminolysis inhibition in colorectal cancer cells.

### Myc mediates the inhibition of NDRG2 in glycolysis and glutaminolysis metabolism of colorectal cancer cells

**Figure 3:**
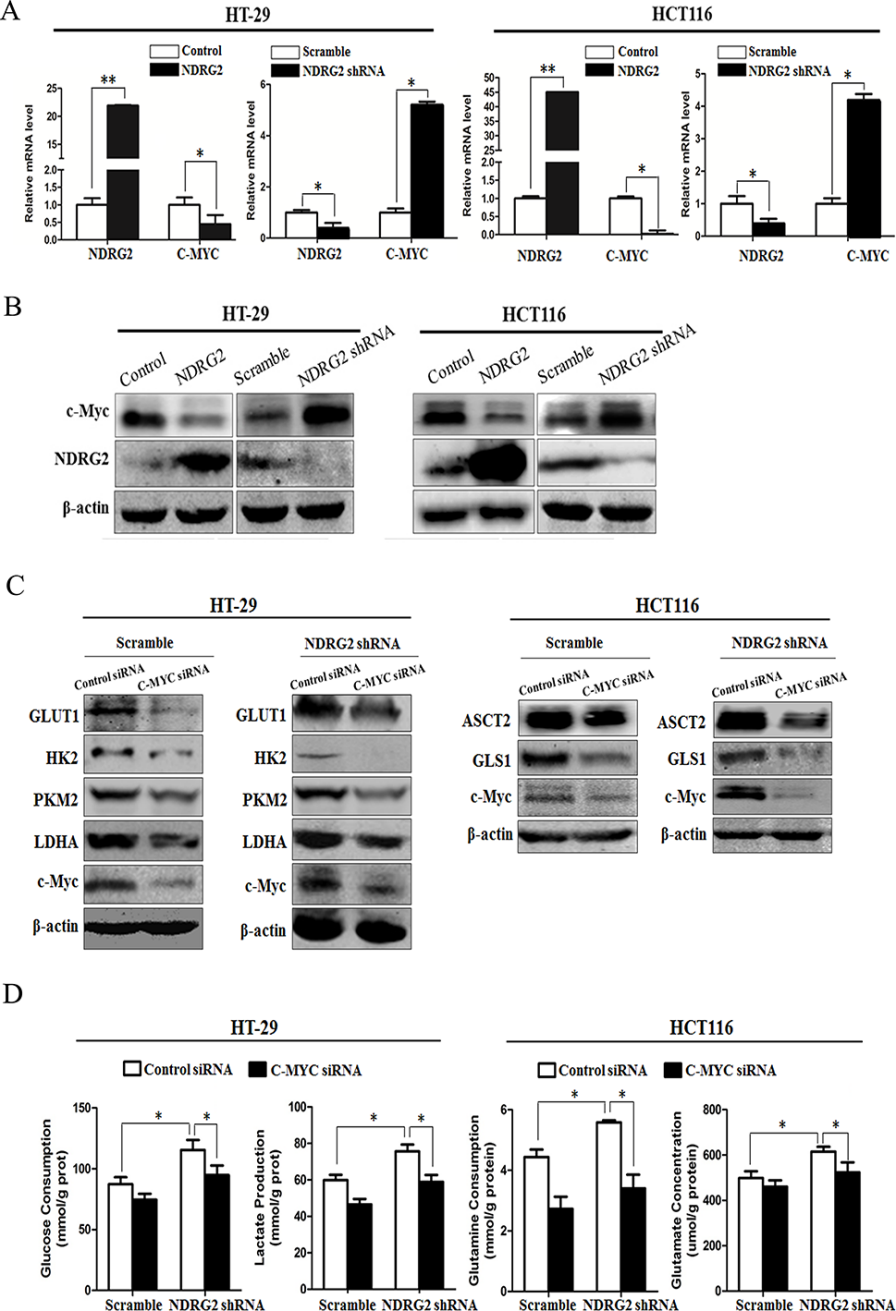
c-Myc mediates NDRG2 inhibition of glycolysis and glutaminolysis in colorectal cancer cells **A.** NDRG2 and C-MYC mRNA levels in HT-29 cells and HCT116 cells infected with lentivirus containing NDRG2 or mCherry and NDRG2 shRNA or control shRNA, and β-actin served as an internal control to ensure equal loading. **B.** NDRG2 and c-Myc protein levels in HT-29 cells and HCT116 cells infected with lentivirus containing NDRG2 or mCherry and NDRG2 shRNA or control shRNA, and β-actin served as an internal control to ensure equal loading. **C.** Protein levels of c-Myc, GLUT1, HK2, PKM2 and LDHA in HT-29 cells infected with lentivirus containing NDRG2 shRNA or control shRNA, and then transfected with C-MYC siRNA or control siRNA. Protein levels of c-Myc, ASCT2 and GLS1 in HCT116 cells infected with lentivirus containing NDRG2 shRNA or control shRNA, and then transfected with C-MYC siRNA or control siRNA. **D.** Glucose consumption, lactate production, glutamine consumption, and glutamate concentration in above-mentioned HT-29 and HCT116 cells. All *P < 0.05; **P < 0.01.

The glucose transporter 1 and key regulatory enzymes in glycolysis, HK2, PKM2 and LDHA, are transcriptionally activated by the oncogenic transcriptional factor c-Myc [6, 10–14]. Meanwhile, glutamine transporter ASCT2 and glutaminase GLS1 are also transcriptionally activated by c-Myc [19]. We therefore analyzed the mRNA and protein expression of c-Myc in NDRG2-overexpressing and NDRG2-knockdown HT-29 and HCT116 cells. Overexpression of NDRG2 consistently decreased c-Myc expression while NDRG2 knockdown increased c-Myc expression in HT-29 and HCT116 cells (Figure 3A and 3B). These data implicated NDRG2 inhibited c-Myc expression in colorectal cancer HT-29 and HCT116 cells.

NDRG2 inhibited glucose transporter 1, and three key regulatory enzymes HK2, PKM2 and LDHA in glycolysis, NDRG2 also inhibited glutamine transporter ASCT2 and glutaminase GLS1 in glutaminolysis, along with the inhibition of c-Myc in cancer cells. We further investigated the role of c-Myc in NDRG2-induced glycolysis and glutaminolysis inhibition in colorectal cancer cells. Endogenous c-Myc in HEK-293T cells were suppressed effectively by transfection with siRNAs (siRNA-1 and siRNA-2) against c-Myc for 48 hours, and siRNA-2 was especially effective at reducing c-Myc expression in HT-29 and HCT116 cells (Supplementary Figure S6A). Furthermore, c-Myc knockdown by siRNA decreased GLUT1, HK2, PKM2 and LDHA expression in NDRG2-knockdown HT-29 cells, which also reduced ASCT2 and GLS1 expression in NDRG2-knockdown HCT116 cells (Figure 3C). In addition, c-Myc knockdown also reduced glucose consumption and lactate production, glutamine consumption and glutamate concentration in the culture medium in the NDRG2-knockdown cells (Figure 3D). Taken together, we have identified that NDRG2 inhibited aerobic glycolysis and glutaminolysis, which were mediated by c-Myc in colorectal cancer cells.

### NDRG2 inhibits c-Myc expression through suppressing β-catenin expression

**Figure 4:**
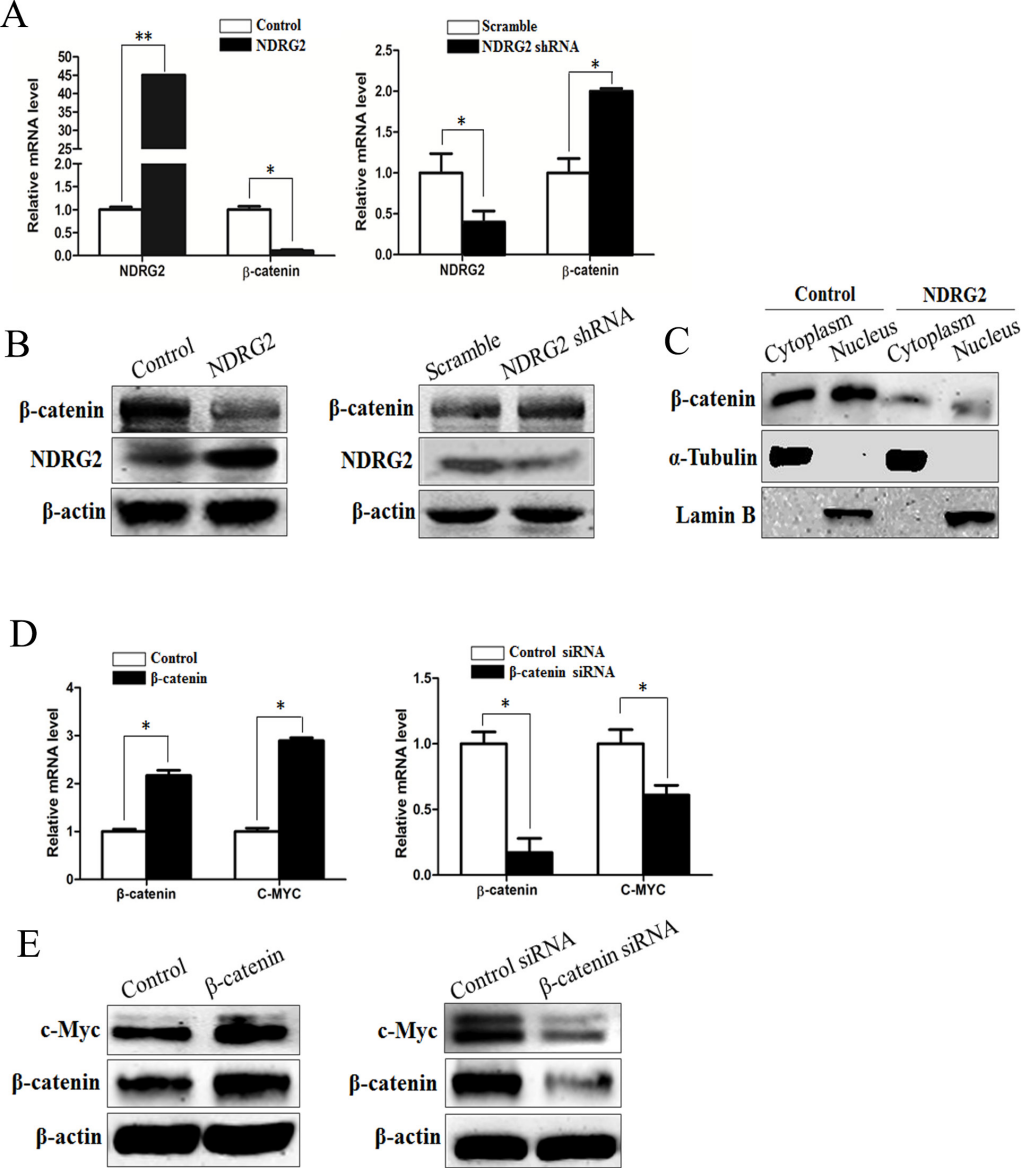
NDRG2 inhibits the expression of β-catenin **A.** The mRNA level of NDRG2 and β-catenin in HCT116 cells infected with lentivirus containing NDRG2 or mCherry and NDRG2 shRNA or control shRNA, and β-actin served as an internal control to ensure equal loading. **B.** The protein level of NDRG2 and β-catenin in HCT116 cells infected with lentivirus containing NDRG2 or mCherry and NDRG2 shRNA or control shRNA, and β-actin served as an internal control to ensure equal loading. **C.** The subcellular distribution of β-catenin in cytoplasm and nucleus of HCT116 cells infected with lentivirus containing NDRG2 or mCherry, α-tubulin and Lamin B served as internal control of cell cytoplasm and nucleus accordingly. **D.** The mRNA level of β-catenin and c-Myc in HCT116 cells transfected with pcDNA3.0 or pcDNA3.0-β-catenin and control siRNA or β-catenin siRNA, and β-actin served as an internal control to ensure equal loading. **E.** The protein level of β-catenin and c-Myc in HCT116 cells transfected with pcDNA3.0 or pcDNA3.0-β-catenin and control siRNA or β-catenin siRNA, and β-actin served as an internal control to ensure equal loading.

β-catenin is the central downstream effector of Wnt signaling pathway and plays an important role in cell proliferation and growth [38]. When Wnt ligand is absent, the level of cytoplasmic β-catenin is regulated by the multiprotein complex of adenomatous polyposis coli (APC), GSK-3β and casein kinase 1α (CK1α), which facilitates proteasomal degradation of β-catenin [39]. However, APC mutation or dysregulation of Wnt ligands results in loss of β-catenin degradation in colorectal cancer cells [40]. Consequently, β-catenin is translocated from cytoplasm to the nucleus, where it binds to the T-cell factor/lymphoid enhancer factor and thereby activates transcription of its target genes such as c-Myc [38]. To evaluate the influence of NDRG2 on β-catenin expression level and subcellular localization, we analyzed the mRNA and protein level of β-catenin in NDRG2-overexpressing and NDRG2-knockdown HCT116 cells, the cytoplasmic and nuclear β-catenin protein level in NDRG2-overexpressing HCT116 cells. Results indicated that the expression level of β-catenin decreased obviously in NDRG2-overexpressing HCT116 cells and increased in NDRG2-knockdown HCT116 cells (Figure 4A and 4B). Accordingly, the cytoplasmic and nuclear β-catenin decreased in NDRG2-overexpressing HCT116 cells (Figure 4C). In addition, the expression level of c-Myc increased in β-catenin-overexpressing HCT116 cells and decreased in β-catenin-knockdown HCT116 cells (Figure 4D and 4E, Supplementary Figure S6B). Briefly, it is indicated that NDRG2 inhibited the expression of β-catenin, and therefore repressed c-Myc expression.

### Correlation between the expression of NDRG2 and c-Myc, β-catenin, GLUT1, HK2, PKM2, LDHA, ASCT2, GLS1 in clinical colorectal carcinomas

**Figure 5:**
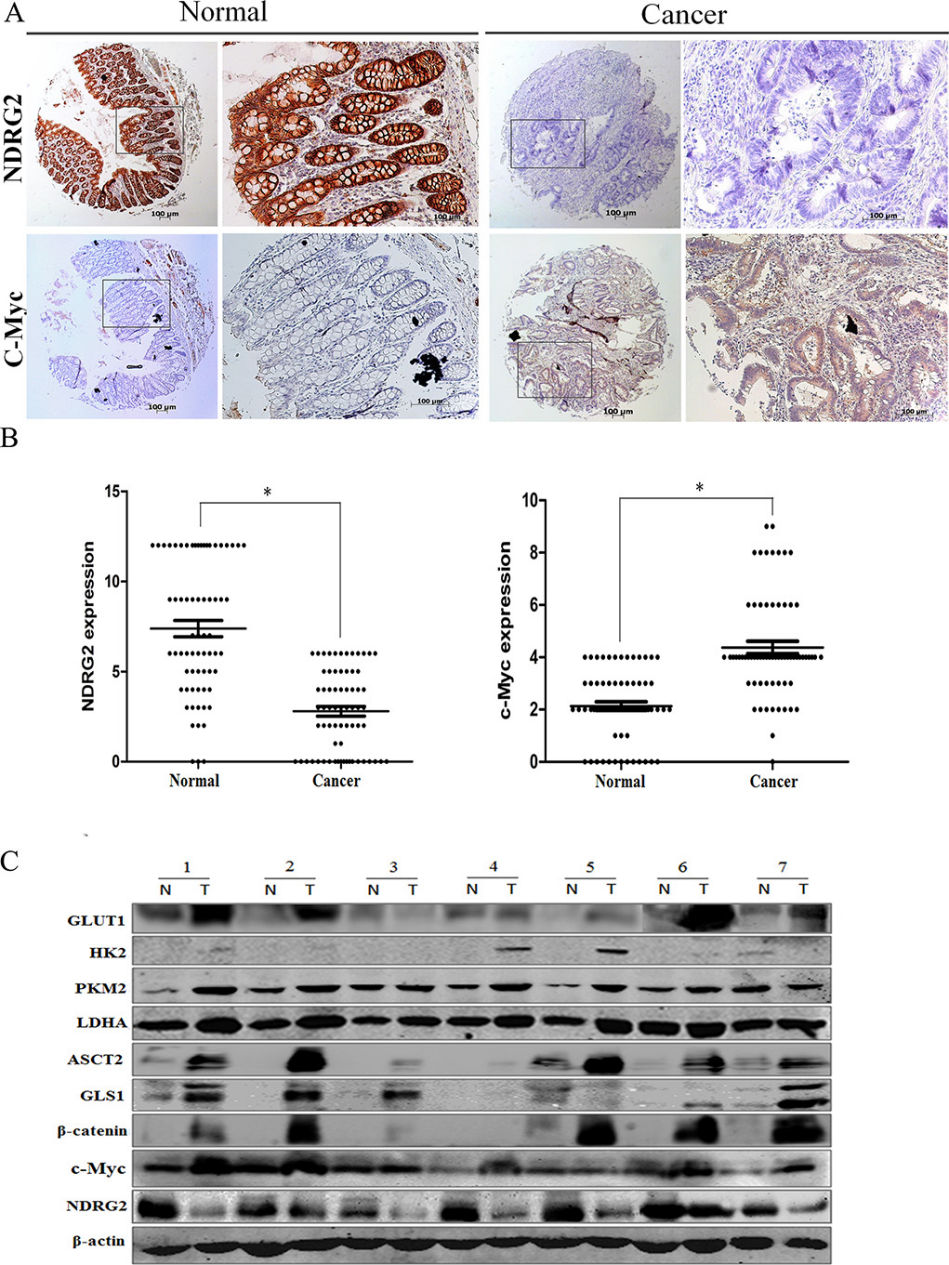
The expression analysis of NDRG2 and metabolism related molecules in clinical colorectal carcinomas **A.** The expression of NDRG2 and c-Myc in human adjacent normal and cancerous colorectal tissue microarrays containing 68 patients was evaluated by immunohistochemistry. Representative photos show the inverse expression of NDRG2 and c-Myc. **B.** Relative expression level of NDRG2 and c-Myc in human colorectal tissue microarrays. Student *t*-test was used for statistical analyses. All *P < 0.05, paired student *t*-test. **C.** The protein level of NDRG2, c-Myc, β-catenin, glucose transporter GLUT1, glycolytic enzymes HK2, PKM2 and LDHA, glutamine transporter ASCT2, glutaminolytic enzyme GLS1 in the fresh colorectal cancer tissues (T) and adjacent normal tissues (N) were detected by western blot, and β-actin served as an internal control to ensure equal loading.

To verify the relationship between NDRG2 and the target molecules *in vivo*, we also investigated the expression of NDRG2 and c-Myc, β-catenin, GLUT1, HK2, PKM2, LDHA, ASCT2, GLS1 in tissues from clinical colorectal carcinomas samples. At first, we analyzed the expression of NDRG2 in tissue microarrays containing 68 colorectal carcinomas and adjacent normal colorectal tissues by immunohistochemistry. Consistent with our previous study, NDRG2 and c-Myc is mainly expressed in the glandular epithelium cells [41]. Furthermore, NDRG2 expression level in colorectal carcinomas tissues was lower than that of adjacent normal tissue, while the expression pattern of c-Myc was the inverse association of NDRG2 (Figure 5A and 5B). Western blot analysis of fresh colorectal tumor and adjacent non-tumor tissues from 7 patients with colorectal cancer revealed that GLUT1, HK2, PKM2, LDHA, ASCT2, GLS1, β-catenin and c-Myc increased significantly in colorectal carcinomas tissues compared with normal colorectal tissues, and there exists a negative correlation between the expression of NDRG2 and target molecules in colorectal tissues (Figure 5C and Supplementary Figure S7). In addition, HK2, PKM2, LDHA, GLS1 also increased significantly in colorectal tissues of *NDRG2* knock-out mouse (Supplementary Figure S8B). These results strongly suggested that NDRG2 inhibited the expression of c-Myc, β-catenin, GLUT1, HK2, PKM2, LDHA, ASCT2, and GLS1 in colorectal cancer.

### NDRG2 inhibits the growth and proliferation of colorectal cancer cells

**Figure 6:**
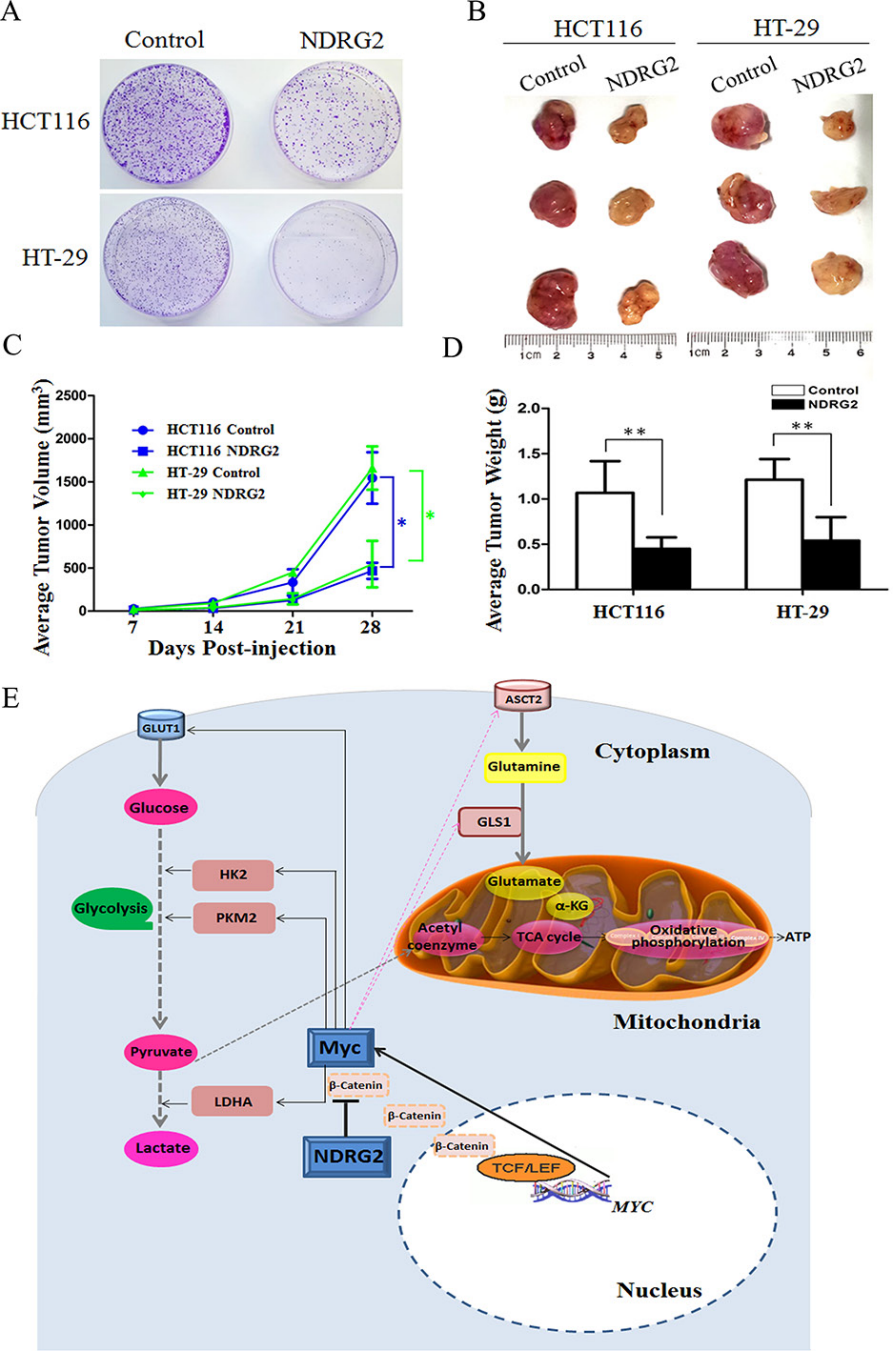
NDRG2 inhibits the growth and proliferation of colorectal cancer cells **A.** Equal numbers of NDRG2-overexpressing HCT116 (HT-29) cells and control cells were seeded onto 60 mm dish. After 14 days, the cells were fixed and stained with crystal violet. **B.** Mice were injected subcutaneously in the right limb with 1 × 10^7^ NDRG2-overexpressing or control HCT116 (HT-29) cells respectively. Representative tumor formation was photographed 28 days after injection. **C.** Tumors volume was calculated by the formula (width^2^ × length × 0.5). **D.** Tumor weight of mice was calculated 28 days after injection. All *P < 0.05; **P < 0.01. **E.** A suggested model for the regulation of metabolic pathways in colorectal cancer cells by tumor suppressor NDRG2. NDRG2 inhibited glycolysis via regulating glucose transporter GLUT1, glycolytic enzymes HK2, PKM2 and LDHA. NDRG2 also inhibited glutaminolysis via regulating glutamine transporter ASCT2 and glutaminase GLS1 in colorectal cancer cells. Transcriptional factor c-Myc mediated inhibition of glycolysis and glutaminolysis by NDRG2. Moreover, NDRG2 inhibited intracellular β-catenin levels and TCF/LEF transcription activity, which lead to the transcriptional inhibition of *C-MYC* gene.

The growth and proliferation of cancer cells rely on glycolysis and glutaminolysis which contribute to ATP production and macromolecule biosynthesis [1]. To determine whether NDRG2 regulate the growth and proliferation of colorectal cancer cells, plate colony formation assays and *in vivo* tumorigenicity assays were performed to evaluate the growth and proliferation ability of NDRG2-overexpressing HCT116 cells and HT-29 cells. Here, we found that NDRG2 overexpression inhibited significantly the colony-forming ability of HCT116 and HT-29 cells (Figure 6A). Moreover, NDRG2 overexpression inhibited tumor growth *in vivo*. The mice injected with HCT116 or HT-29 cells expressing NDRG2 developed tumors more slowly than the control groups (Figure 6B). By 4th week, mice injected with NDRG2-overexpressing HCT116 or HT-29 cells showed a statistically significant decrease in average tumor volume compared with the control groups (Figure 6C). As a result, the average tumor weight in mice injected with NDRG2-overexpressing HCT116 or HT-29 cells was significantly less than that of the control cells (*P* < 0.01, Figure 6D). These data demonstrate that NDRG2 suppresses the growth and proliferation of colorectal cancer cells *in vitro* and *in vivo*.

## DISCUSSION

Tumor metabolism reprogramming, which fuels limitless tumor replication and growth, was added to the new hallmark of cancer [37]. However, the molecular mechanisms leading to the metabolic property of tumors are not well defined. In this study, we discovered that tumor suppressor NDRG2 participates in tumor metabolism, especially in glycolysis and glutaminolysis. Our previous research revealed that NDRG2, which was discovered by our research group, inhibited tumor cell proliferation [23, 32] and invasion [35, 42]. It is also reported that NDRG2 inhibits glucose uptake and breast tumor growth by promoting GLUT1 protein degradation without affecting GLUT1 transcription [43]. Our current experiments demonstrate for the first time that NDRG2, which is downregulated in colorectal cancer, inhibits glycolysis and glutaminolysis in addition to inhibiting cancer cell proliferation and invasion. Furthermore, NDRG2 inhibits the expression of glucose transporter GLUT1 and glycolytic key enzymes HK2, PKM2 and LDHA, the expression of glutamine transporter ASCT2 and glutaminolytic enzyme GLS1 at the transcriptional level. The transcription factor c-Myc, which is inhibited by NDRG2, mediates the inhibition of glycolysis and glutaminolysis induced by NDRG2. More importantly, NDRG2 inhibited the expression of c-Myc by suppressing the expression of β-catenin, which can activate the transcription of c-Myc after shuttling into the nucleus [44]. These findings provide evidence that NDRG2 inhibits tumor glycolysis and glutaminolysis, and additional in-depth studies on the role of NDRG2 in other tumor glucose catabolism pathways and tumor fatty acid metabolism are needed.

The proto-oncogene *MYC* encodes the transcription factor c-Myc, which links altered cellular metabolism to tumorigenesis. Transcription factor c-Myc regulates glucose and glutamine metabolism in addition to the biogenesis of ribosomes and mitochondria [6]. As a key transcription factor, many glucose and glutamine metabolism genes can be directly regulated by c-Myc, such as glucose transporter 1 (GLUT1), hexokinase 2 (HK2), pyruvate kinase M2 isoform (PKM2), lactate dehydrogenase A (LDHA), glutamine transporter (ASCT2) and glutaminase 1 (GLS1) [6]. In the present study, we have investigated the expression of multiple glucose transporters, glutamine transporters, catalytic enzymes involved in glycolysis and glutaminolysis pathway in NDRG2-overexpressing and knockdown colorectal cancer cells (Supplementary Figure S9), and we have also detected PTEN/Akt signaling pathway in NDRG2-overexpressing colorectal cancer cells (Supplementary Figure S10). Our research data revealed that only GLUT1, HK2, PKM2, LDHA, ASCT2 and GLS1 were significantly inhibited by NDRG2, and these metabolic targets regulated by NDRG2 in glycolysis and glutaminolysis are consistent with that of c-Myc. Our subsequent study confirmed that NDRG2 inhibited c-Myc expression and c-Myc mediated tumor glycolysis and glutaminolysis reprogramming.

Amplification of oncogene c-myc has been described in primary colorectal tumors, and c-myc overexpression was observed in 60% of patients with colorectal cancer [45]. In our results, c-Myc expression increased in colorectal carcinomas tissues compared with normal colorectal tissues (Supplementary Figure S7). However, our results also showed c-Myc expression decreased in one sample of colorectal carcinomas tissues (Figure 5C). In fact, overexpress c-myc was offset when c-myc overexpression was coupled with a point mutated p53 gene in patients. Therefore, c-myc deregulation may be regulated by a more downstream event such as point mutation of p53 gene in colorectal adenocarcinoma [46]. The evaluation of c-Myc expression in clinical colorectal carcinomas tissues needs to be studied in the context of a more complex, multigenic environment.

ASCT2 is an important glutamine transporter, and influences metabolic and signaling function of glutamine and glutamate. It is highly expressed in human colorectal cancer cell lines Caco-2, HT-29, HCT116, and LS 174T. However, the expression of ASCT2 is very low in mouse colorectal cancer cell line CT26 and mouse colon tissues, as well as in mouse kidney tissues (Supplementary Figure S8A). Maybe due to the differences in metabolic rate and dietary composition, the expression abundance of ASCT2 in human is much higher than that in mouse. In addition, the expression of GLUT1 decreases obviously in NDRG2 knock-out mouse (Supplementary Figure S8B), along with the compensatory increase of NDRG1 (Supplementary Figure S8C). The correlation between GLUT1 and NDRG1 in mouse embryonic development need to be further studied.

Our previous study reported that human NDRG2 was transcriptionally repressed by c-Myc [47]. Interestingly, our present study found that NDRG2 also repressed the expression of c-Myc at transcriptional level through inhibiting the expression of β-catenin. It is reported that nuclear β-catenin can bind to the T-cell factor/lymphoid enhancer factor (TCF/LEF) and thereby activates transcription of its target genes such as c-Myc [38]. Our preliminary research work indicates that NDRG2 may regulate T-cell factor (TCF)/β-catenin signaling pathway, which lead to the inhibition of many target genes including *C-MYC* in human colon carcinoma (data not shown), and the related regulation mechanism is being investigated by our research group.

Recent study points out that NDRG2 can reduce the phosphorylation of AMPK, and acts as a negative regulator of LKB1-AMPK pathway [48]. Interestingly, there exists crosstalk between LKB1-AMPK pathway and β-catenin, knockdown of LKB1 can preserve head and neck squamous cancer cells (HNSCC) against β-catenin silencing-induced cytotoxicity [49]. Whether LKB1-AMPK pathway is involved in the inhibition of NDRG2 on β-catenin expression requires further investigation.

In summary, this study illustrates the regulatory role and molecular mechanism of tumor suppressor NDRG2 in metabolic reprogramming of colorectal cancer. The ability of NDRG2 to inhibit glycolytic and glutaminolytic targets contributes to the repression of c-Myc, leading to inhibition of colorectal cancer cells' growth and proliferation. We propose a scheme (Figure 6E) for NDRG2-mediated glycolysis and glutaminolysis inhibition of colorectal cancer. With these discoveries, we could further understand the mechanism of tumor metabolic reprogramming and provide a novel metabolic target for tumor treatment.

## MATERIALS AND METHODS

### Tumor tissue samples

Fresh colorectal tumor and adjacent non-tumor tissues from 7 patients with colorectal cancer were obtained from Xijing Hospital of Digestive Diseases with informed consent and approval from the Clinical Research Ethics Committee of Xijing Hospital, the Fourth Military Medical University. Tissue microarrays from 68 patients with colorectal cancer were obtained from the Department of Pathology of Xijing Hospital. In each case, a diagnosis of primary colorectal cancer was performed. Information on patient age, gender, size of primary tumor, tumor differentiation, T classification, N classification was collected and represented are summarized in Table 1.

**Table 1:** Characteristics of the patients in the tissue microarrays cohort according to the cancer status, NDRG2 and c-Myc expression

Patients demographics	Total *N* = 68	NDRG2		*P^a^*	c-Myc		*P^a^*
		Negative	Positive		Negative	Positive	
*Sex*				0.821			0.846
Male	38	11	27		1	37	
Female	30	9	21		0	30	
*Age of diagnosis, years*				0.835			0.866
< 60	35	12	23		0	35	
> 60	33	8	25		1	32	
*Differentiation status*				< 0.001			0.006
Poor	5	4	1		0	5	
Moderate	48	16	32		0	48	
Well	15	0	15		1	14	
*TNM stage*				0.547			0.332
I	1	0	1		0	1	
II	6	2	4		1	5	
III	48	14	34		0	48	
IV	13	4	9		0	13	

Abbreviation: NDRG2, N-Myc downstream-regulated gene 2; TNM, tumor node metastasis.

### Cell cultures

Colorectal cancer cell lines HT-29, Caco-2, LS 174T, HCT116 and human embryonic kidney cell line HEK-293T were purchased from American Type Culture Collection (Manassas, VA, USA). The HT-29 and HCT116 cells were cultured in McCoy's 5A Modified Medium, Caco-2, LS 174T and HEK-293T cells were cultured in Dulbecco's modified Eagle's Medium (DMEM), supplemented with 10% fetal bovine serum respectively, in a humidified atmosphere of 5% CO_2_ at 37°C.

### Plasmid construction, virus packaging, and infection

Recombinant lentiviral vectors were constructed with Invitrogen ViraPower™ Lentiviral System (Carlsbad, CA, USA) in our laboratory. Full-length DNA of human NDRG2 were cloned and subcloned into the vector pLenti6. Short hairpin RNAs (shRNA) against human NDRG2 were designed to mediate gene silencing and were subcloned into the EcoR I/Age I sites of pLKO-TRC vector. The shRNA sequences specific for NDRG2 are shown in Supplementary Table S1. HEK-293T cells were transfected with pLenti6-mCherry/NDRG2, pLKO-Scramble/NDRG2-shRNA, PAX2 and PMD2G lentiviral vectors using Lipofectamine 2000 (Invitrogen) according to the manufacturer's instructions. After 48 h, the lentiviral supernatants were collected, filtered (0.45-μm size filter; Millipore, Billerica, MA, USA), then added into the HT-29, Caco-2, LS 174T and HCT116 cells. Human β-catenin in the pcDNA3.0 vector was obtained from Addgene (Cambridge, MA, USA).

### Gene transfection

HT-29 and HCT116 cells were seeded in 6-well plates at a density of 5 × 10^5^ cells/well. Cells were transfected with 60 nM small interfering RNA (Gene-Pharma, Shanghai, China) or the indicated plasmids using Lipofectamin 2000 (Invitrogen), according to the manufacturer's protocol. The c-Myc and β-catenin siRNAs sequences are shown in Supplementary Table 2. Cells were exposed to siRNA or plasmid for 6 h, after which the medium replaced with McCoy's 5A Medium and the cells were incubated for 48 h.

### Quantitative real-time PCR and western blot analysis

Total RNA was isolated from cells using Trizol Reagent (Invitrogen), and then complementary DNA (cDNA) was synthesized using AMV reverse transcriptase (Promega, Madison, WI, USA) according to the manufacturer's instructions. The cDNA was used as a template for quantitative real-time PCR using the ABI Prism 7500 real-time PCR instrument (Applied Biosystems, Carlsbad, CA, USA). The primers used in real-time quantitative PCR are shown in Supplementary Table S3.

For Western blot analysis, total protein was prepared from cell lines and clinical colorectal carcinomas tissue samples. Immunoblotting were performed according to standard procedures with polyclonal rabbit anti-human HK2, PKM2, LDHA, ASCT2, c-Myc, β-catenin (Cell Signaling, Bedford, MA, USA), polyclonal rabbit anti-human GLS1 (Abcam, Cambridge, UK), monoclonal mouse anti-human GLUT1 (Abcam, Cambridge, UK), monoclonal mouse anti-human NDRG2 (Abnova, Taipei, Taiwan), and polyclonal rabbit anti-human β-actin (Biosynthesis Biotechnology, Beijing, China) antibody.

### Glucose consumption and lactate production

Treated cells were seeded on 6-well plates at a density of 1 × 10^5^ cells per well and the culture medium was changed to DMEM after incubation overnight. The concentrations of glucose and lactate in culture medium were measured after 24 h with Glucose Test Kit (Invitrogen) and Lactate Assay Kit (Jiancheng Bioengineering, Nanjing, China) individually.

### Glucose transport assay

Glucose transport was measured using the glucose analog 2-NBDG (Molecular Probes, Eugene, OR) as a fluorescent probe. To evaluate the kinetics of 2-NBDG uptake, equal number of cells were seeded in 6-well plates at a density of 1 × 10^5^ cells per well. After 48 h, the cells were pre-incubated in glucose-free Krebs-Ringer bicarbonate (KRB) buffer for 15 min and then incubated in fresh KRB buffer supplemented with 600 μM 2-NBDG, a D-glucose fluorescent analogue, for 30 min at 37°C in a humidified atmosphere of 5% CO_2_. The stained cells were quantitatively analyzed by flow cytometry using a FACSCalibur™ flow cytometer (Beckton Dickinson).

### Enzyme activity

Cells were lysed in isolation buffer provided in corresponding assay kit, and the activities of metabolic enzymes HK, PYK and LDH were determined by the corresponding assay kits according to the manufacturer's recommendation (Jiancheng Bioengineering, Nanjing, China) individually. Total enzyme activity was normalized to the protein content of the cell lysate.

### Glutamine/glutamate concentration

Cells in a 6 well plate were cultured for 24 h in medium without phenol-red. The culture medium was collected and cells were lysed with RIPA buffer. Concentrations of glutamine in the medium and in the cell lysate were determined with the glutamine/glutamate determination kit (GLN-1; Sigma-Aldrich). Each sample was divided into two parts: part 1 was measured with glutaminase for transferring the glutamine into glutamate, part 2 was measured directly. Samples were then dehydrogenized to α-ketoglutarate accompanied by reduction of NAD+ to NADH. The amount of NADH is proportional to the amount of glutamate and was measured using a spectrophotometer at 340 nm. A standard curve was determined for each experiment to calculate the concentration of glutamate in samples. Glutamine levels were calculated (part 1 minus part 2) and normalized to total protein levels. The glutamine level of normal culture medium was also measured, and the glutamine consumption was calculated (as glutamine level in normal medium minus glutamine level in medium after culturing cells) and normalized to protein level.

### Immunohistochemistry

TMA staining was performed by standard immunohistochemistry procedures. The slides were incubated overnight using primary antibodies against NDRG2 (Abnova, Taipei, Taiwan) or c-Myc (Boster, Wuhan, China). Mayer's hematoxylin was used for the purpose of nuclear counter staining. In this study, the number of positively stained cells and the intensity of positive staining on epithelium cells were independently scored by two pathologists in a blinded manner. The extensional immunoreactivity score standard used (1) the number of cells with positive staining (≤5%: 0; 6–25%: 1; 26–50%: 2; 51–75%: 3; and >75%: 4) and (2) the staining intensity (colorless: 0; pallide-flavens: 1; yellow: 2; brown: 3). The staining grade was stratified as absent (0 score), weak (1–4 score), moderate (5–8 score) or strong (9–12 score). Tumors with weak, moderate or strong immunostaining intensity were classified as staining positive (+), whereas tumors with no immunostaining were classified as staining negative (−).

### Colony formation assay

Cells were seeded into 60 mm dishes at a density of 200 cells per dish. The cells were grown for 2 weeks in culture medium. Then, the colonies were fixed and stained with crystal violet.

### *In vivo* tumorigenicity assay

Four to six-week-old athymic mice were injected subcutaneously in the right limb with 1 × 10^7^ cells. Tumor growth was monitored by measuring tumor size using Vernier calipers every week for a 4-week period and calculating tumor volume using a standard formula: tumor volume (mm^3^) = width (mm^2^) × length (mm) × 0.5. At the end of the experiment, tumor weight was assessed by sacrificing the mice, removing and weighting the tumor.

### Statistical analysis

Statistical analyses were completed using SPSS 19.0 software (IBM) for Windows. All data shown are mean ± SEM of triplicate values from three separate experiments. *P* < 0.05 was considered to be statistically significant. Independent Student *t*-test or one-way ANOVA were used to compare the continuous variables between the two groups or more than two groups.

## SUPPORTING INFORMATION



## Abbreviations

NDRG2: N-Myc downstream regulated gene 2
cDNA: complementary DNA
DMEM: Dulbecco's modified Eagle medium
ECAR: extracellular acidification rates
OCR: oxygen consumption rates
G6P: glucose-6-phosphate
GLUT1: glucose transporter 1
HIF-1: hypoxia-inducible factor 1
HK2: hexokinase 2
LDHA: lactate dehydrogenase A
Myc: v-myc myelocytomatosis viral oncogene homolog (avian)
NADH: monodehydroascorbate reductase
PYK: pyruvate kinase
PKM2: M2 isoform of pyruvate kinase
GLS1: glutaminase 1
ASCT2: alanine-serine-cysteine (ASC) amino-acid transporter 2
PTEN: phosphatase and tensin homolog
shRNA: short hairpin RNA
siRNA: small interfering RNA
SP: specificity protein
TCF: T-cell factor
LEF: lymphoid enhancing factor
TSC1/2: tuberous sclerosis complex
2-NBDG: 2-[N-(7-nitrobenz-2-oxa-1,3-diaxol-4-yl)amino]-2-deoxyglucose
