# Supplementary material for: Tumor suppressor NDRG2 inhibits glycolysis and glutaminolysis in colorectal cancer cells by repressing c-Myc expression
Source: Oncotarget. 2015 Jul 20;6(28):26161–76. doi: 10.18632/oncotarget.4544 (PMC4694893; doi:10.18632/oncotarget.4544)
Supplement: Supplementary file 1 [file oncotarget-06-26161-s001.pdf]

SUPPORTING INFORMATION

Summary: In supplemental materials and methods, we provide the primers used in this paper, the sequences used to construct shRNAs, and the siRNA sequences targeting C-MYC and β-catenin in Supplementary Tables. We also provide the metabolomics analysis of NDRG2-overexpressing colorectal cancer cells HCT116, the ECAR and OCR in NDRG2-overexpressing HCT116 cells, changes of glycolysis and glutaminolysis in NDRG2-knockdown HCT116 cells transfected with NDRG2 shRNA-1 and NDRG2 shRNA-2, ^{18}F-FDG PET/CT imaging of mice transfected with NDRG2-overexpressing HT-29 cells, the expression level of ASCT2 and GLS1 in LoVo cells infected with lentivirus containing NDRG2 or NDRG2 shRNA, c-Myc and β-catenin knockdown efficiency in HET-293T cells, densitometric analysis of the results of Western Blot that detected the expression of NDRG2 and metabolism-related molecules in clinical colorectal carcinomas, the protein level of ASCT2 in human and mouse colorectal cancer cells, colon and kidney tissues of NDRG2 knock-out mouse, the protein level of NDRG1 in colon tissues of NDRG2 knock-out mouse, the mRNA levels of metabolism-related molecules in NDRG2-overexpressing and NDRG2-knockdown HCT116 cells, the expression and phosphorylation level of PTEN and Akt in NDRG2-overexpressing colorectal cancer cells in Supplementary Figures.

SUPPLEMENTARY MATERIALS AND METHODS

Materials

Human colorectal cancer cell lines HT-29, Caco-2, HCT116, LS 174T, LoVo, mouse colorectal cancer cell line CT26, and human HEK-293T cell line were purchased from American Type Culture Collection (Manassas, VA, USA). The HT-29 and HCT116 cells were cultured in McCoy's 5A Modified Medium, Caco-2, LS 174T and HEK-293T cells were cultured in Dulbecco's modified Eagle's Medium (DMEM), LoVo cells were cultured in Kaighn's modification of Ham's F-12 (F-12K) Medium, CT26 cells were cultured in RPMI1640 Medium, supplemented with 10% fetal bovine serum respectively, in a humidified atmosphere of 5% CO_2 at 37°C. All cell culture reagents were obtained from Gibco BRL Technology.

Metabonomics analysis

Metabolomic profiles were obtained to assess the relative distribution of various metabolites of mCherry-overexpressing colorectal cancer cells (HCT116-Control) and NDRG2-overexpressing colorectal cancer cells (HCT116-NDRG2). Cells were collected and quickly frozen. Further sample preparation, Metabonomics analysis, peak identification and curation were performed by Sensichip (Sensichip Infortech, Shanghai, China).

Extracellular acidification rate and oxygen consumption rate measurement

Extracellular acidification rate (ECAR) and oxygen consumption rate (OCR) were measured in real time with use of a Seahorse Bioscience XF24 extracellular flux analyzer (Seahorse Bioscience, North Billerica, MA). On the day before the experiment, the sensor cartridge was placed in the calibration buffer supplied by Seahorse Bioscience to hydrate overnight. After optimization of cell number, cells were seeded in XF 24-well microplates (40,000/well for HCT116). After an overnight incubation, culture media was exchanged for base media (DMEM supplemented with 1 mM sodium pyruvate) 1 hour before the assay, and the rates (ECAR and OCR) were detected according to the manufacturer's instruction.

^{18}F-FDG PET/CT Imaging

^{18}F-FDG PET/CT was performed using a NanoPET/CT scanner (Mediso, Hungary; Bioscan, USA), whole-body PET/CT was scanned ~60 min after the intravenous injection of ~150 μCi/kg of ^{18}F-FDG. To evaluate ^{18}F-FDG accumulation, Standardized Uptake Value (SUVmean) was measured.

RT-PCR

Total RNA was isolated from cells using Trizol Reagent (Invitrogen), and then complementary DNA (cDNA) was synthesized using AMV reverse transcriptase (Promega, Madison, WI, USA) according to the manufacturer's instructions. The cDNA was used as a template for quantitative real-time PCR assay using the ABI Prism 7500 real-time PCR instrument (Applied Biosystems, Carlsbad, CA, USA) and PCR assay. The primers used in real-time quantitative PCR and PCR are shown in Supplementary Table S3 and Supplementary Table S4.

Western blot analysis

Total protein was prepared from cell lines and mouse tissues, and Western blot analysis were performed according to standard procedures with polyclonal rabbit anti-human HK2, PKM2, LDHA, ASCT2, c-Myc, β-catenin, PTEN, phospho-Akt (Ser473), Akt (Cell Signaling, Bedford, MA, USA), polyclonal rabbit anti-human GLS1 (Abcam, Cambridge, UK), monoclonal mouse anti-human GLUT1 (Abcam, Cambridge, UK), monoclonal mouse anti-human NDRG2 (Abnova, Taipei, Taiwan), polyclonal goat anti-human NDRG1 (Santa Cruz Biotechnology, CA, USA), and polyclonal rabbit anti-human β-actin (Biosynthesis Biotechnology, Beijing, China) antibody.

SUPPLEMENTARY FIGURES AND TABLES

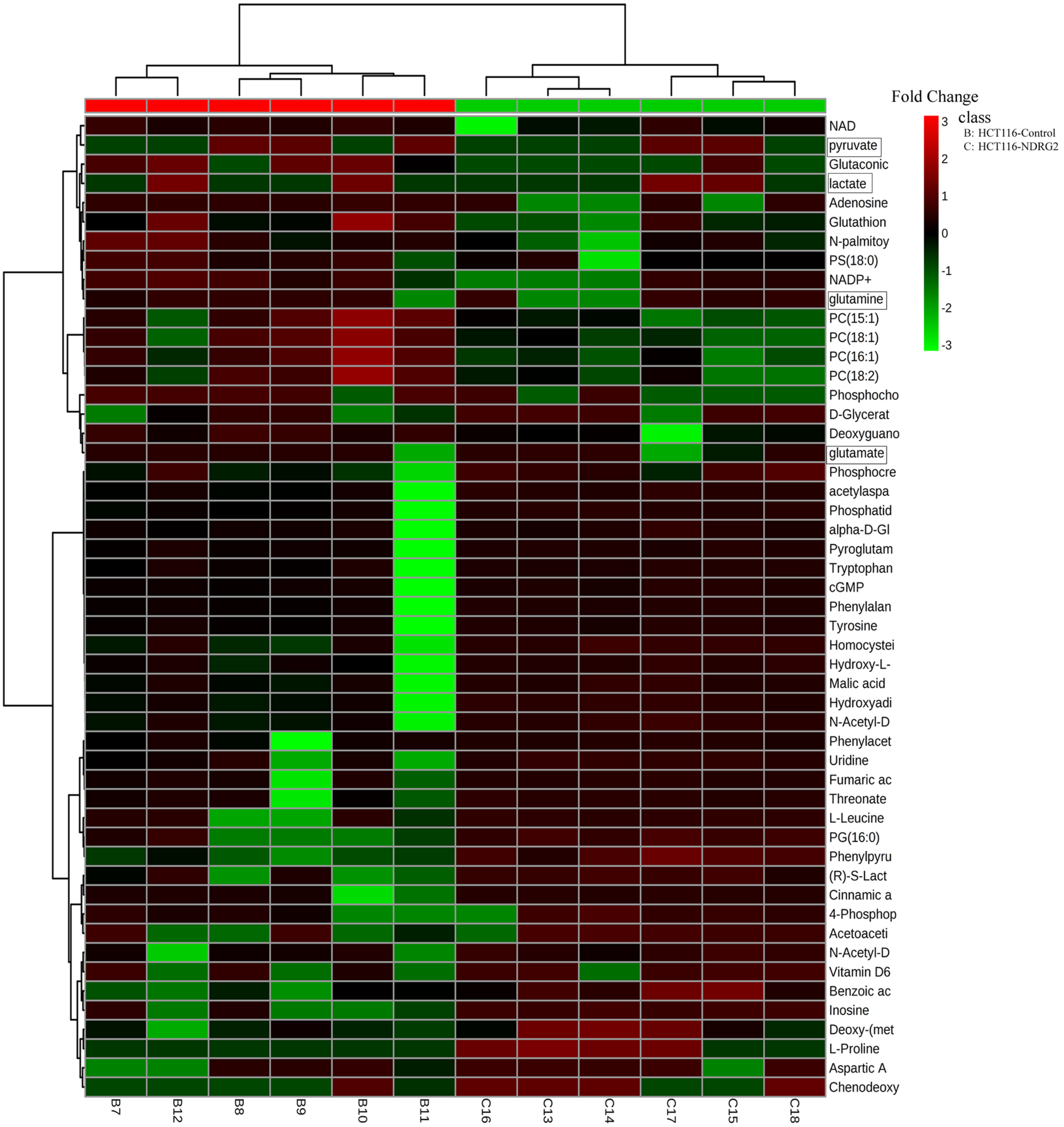

Supplementary Figure S1: NDRG2 changes the metabolic profile and inhibits glycolysis and glutaminolysis in human colorectal cancer cells. Heat map shows biochemical factors in lysates from six replicates each of mCherry-overexpressing colorectal cancer cells HCT116 (HCT116-Control) and NDRG2-overexpressing colorectal cancer cells HCT116 (HCT116-NDRG2). The relative fold change for each biochemical factor in each sample is represented as a relative mean value increase (red) or decrease (green).

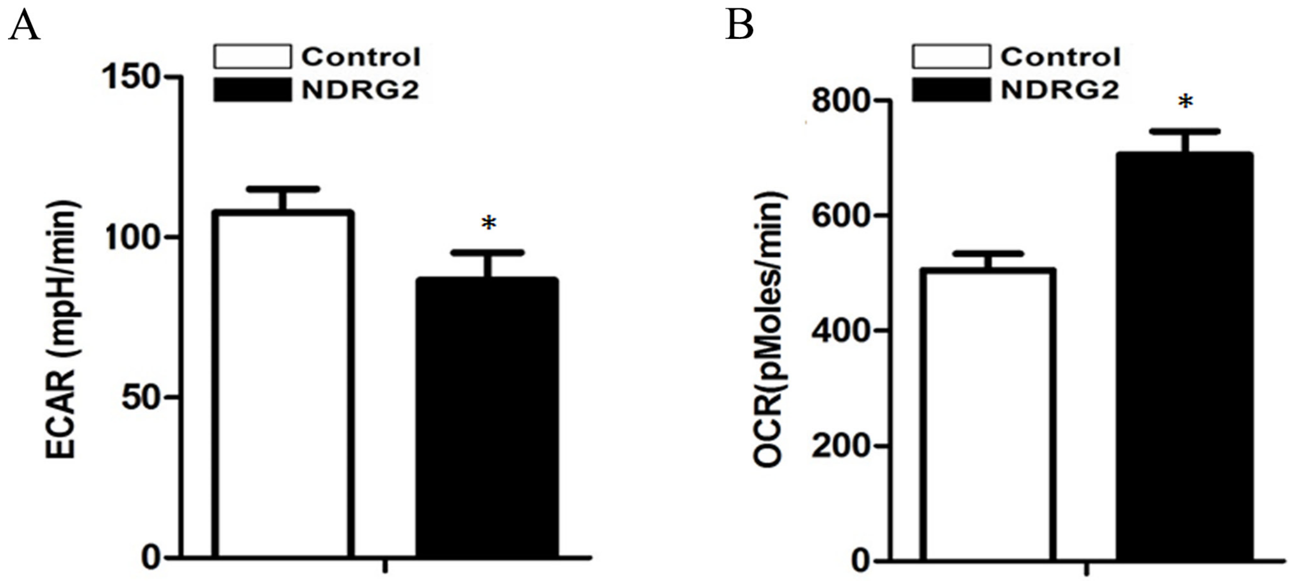

Supplementary Figure S2: NDRG2 inhibits lactate production in human colorectal cancer cells. A. Extracellular acidification rates (ECAR) and **B.** Oxygen consumption rates (OCR) were measured in HCT116 cells infected lentivirus containing NDRG2 or mCherry. *, $P < 0.05$, compared with respective control. Bars indicate mean ± SEM.

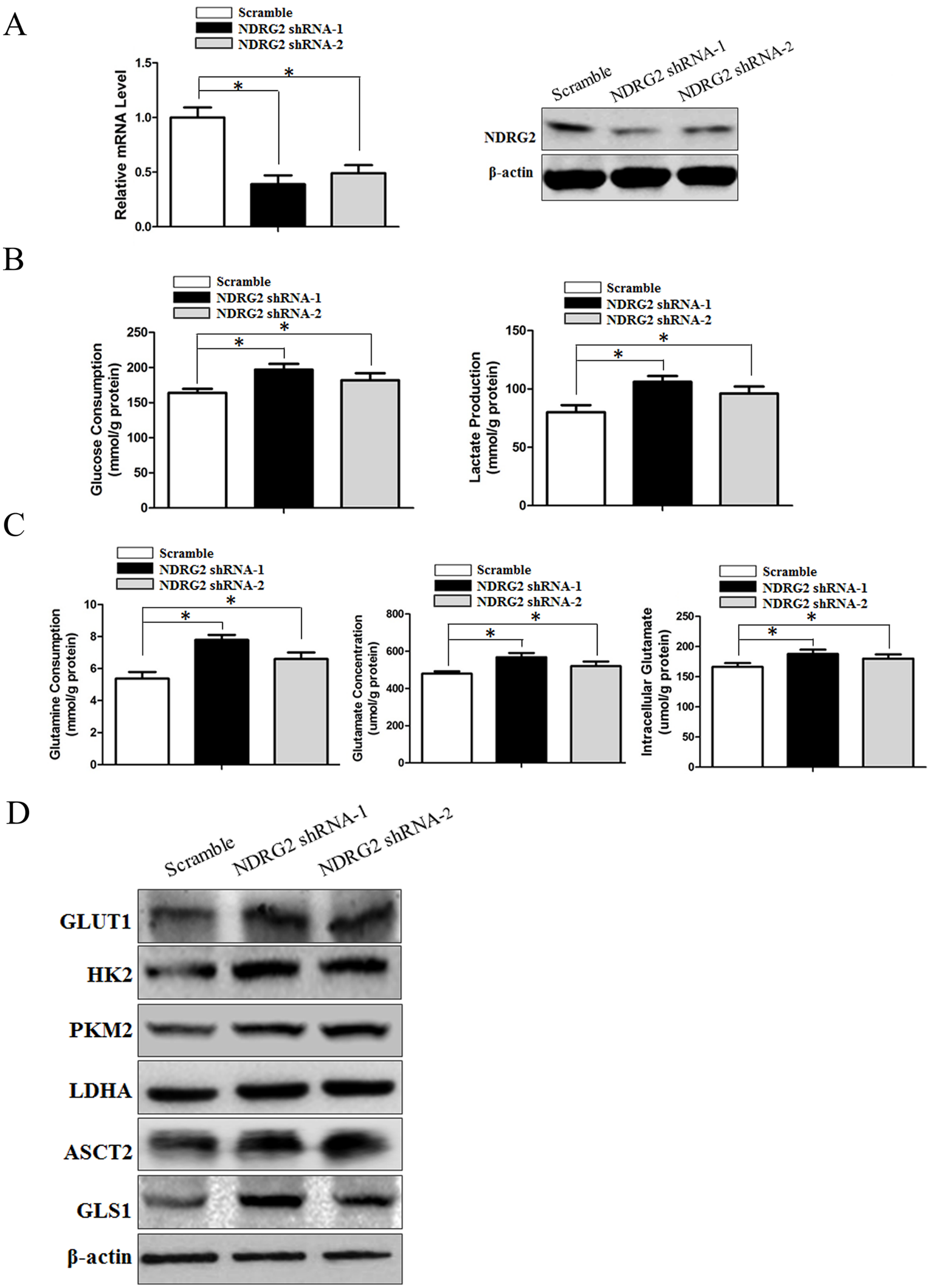

Supplementary Figure S3: Knockdown of NDRG2 promotes glycolysis and glutaminolysis in human colorectal cancer cell line HCT116. A. Quantified NDRG2 mRNA levels and protein levels in HCT116 cells infected with lentivirus containing NDRG2 shRNA-1, NDRG2 shRNA-2 or control shRNA, and β-actin served as an internal control to ensure equal loading. **B.** Glucose consumption and lactate production in NDRG2-knockdown HCT116 cells were assessed. **C.** Glutamine consumption, glutamate concentration and intracellular glutamate in NDRG2-knockdown HCT116 cells were assessed. **D.** GLUT1, HK2, PKM2, LDHA, ASCT2 and GLS1 protein levels in HCT116 cells infected with lentivirus containing NDRG2 shRNA-1, NDRG2 shRNA-2 or control shRNA, and β-actin served as an internal control to ensure equal loading. *, $P < 0.05$, compared with respective control. Bars indicate mean ± SEM.

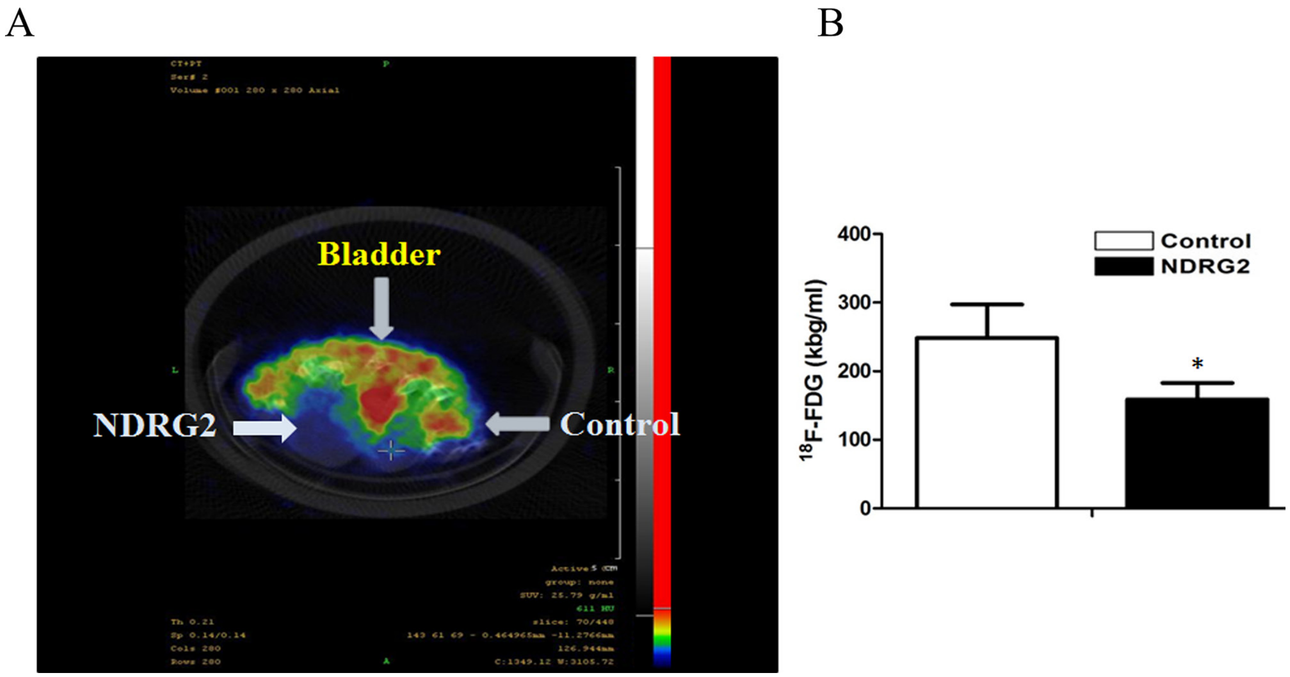

Supplementary Figure S4: NDRG2 inhibits the ^{18}F-FDG uptake in human colorectal cancer cells. A. MicroPET demonstrates that NDRG2 overexpression inhibits ^{18}F-FDG uptake in mice bearing transfected HT-29 cells. **B.** The ^{18}F-FDG uptake was quantified over time using a γ-radiation counter. *, $P < 0.05$, compared with respective control. Bars indicate mean ± SEM.

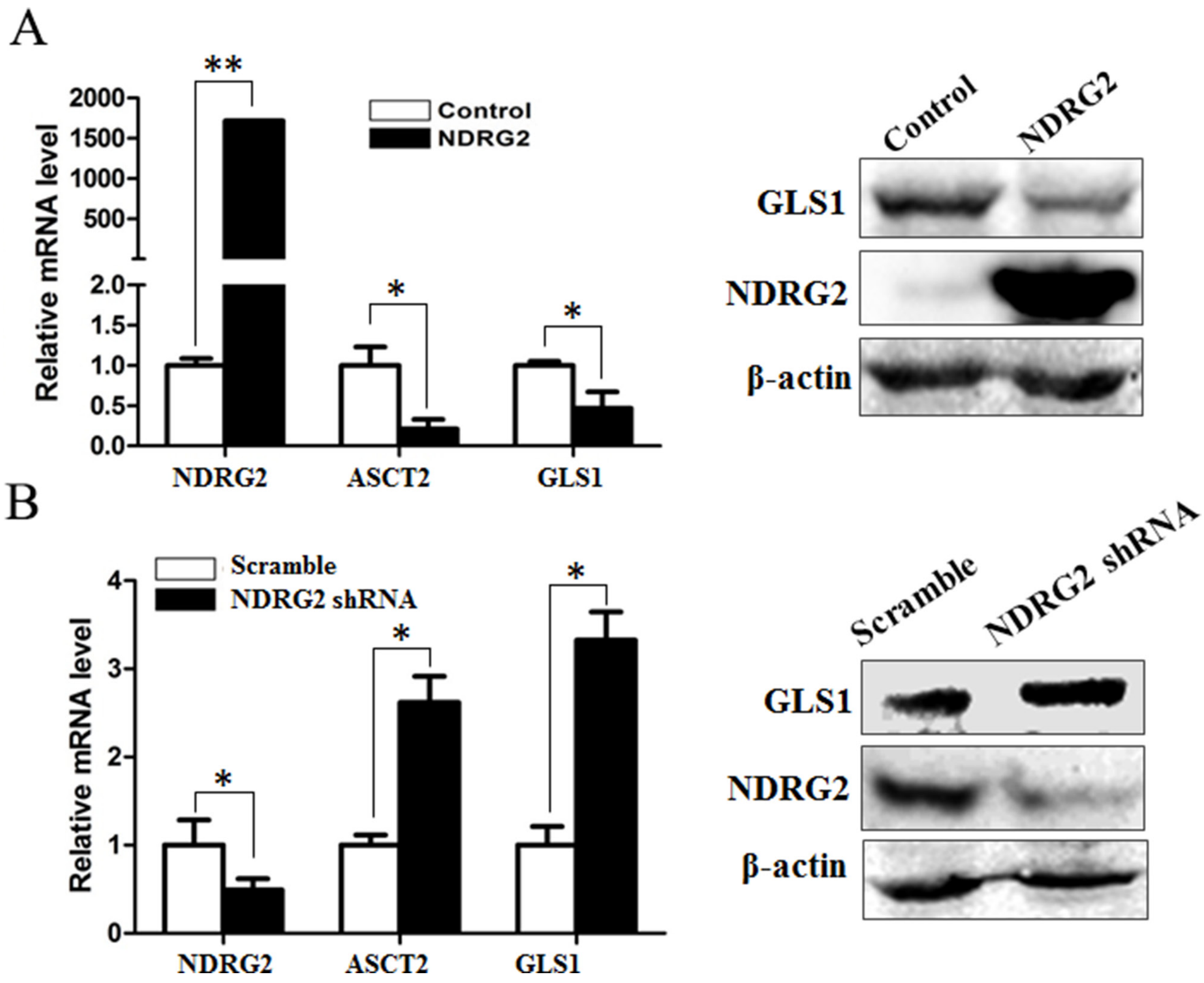

Supplementary Figure S5: NDRG2 inhibits the expression of ASCT2 and GLS1 in human colorectal cancer cells. **A.** NDRG2, ASCT2 and GLS1 mRNA levels, NDRG2 and GLS1 protein levels in LoVo cells infected with lentivirus containing NDRG2 or mCherry, and β-actin served as an internal control to ensure equal loading. **B.** NDRG2, ASCT2 and GLS1 mRNA levels, NDRG2 and GLS1 protein levels in LoVo cells infected with lentivirus containing NDRG2 shRNA or control shRNA, and β-actin served as an internal control to ensure equal loading.

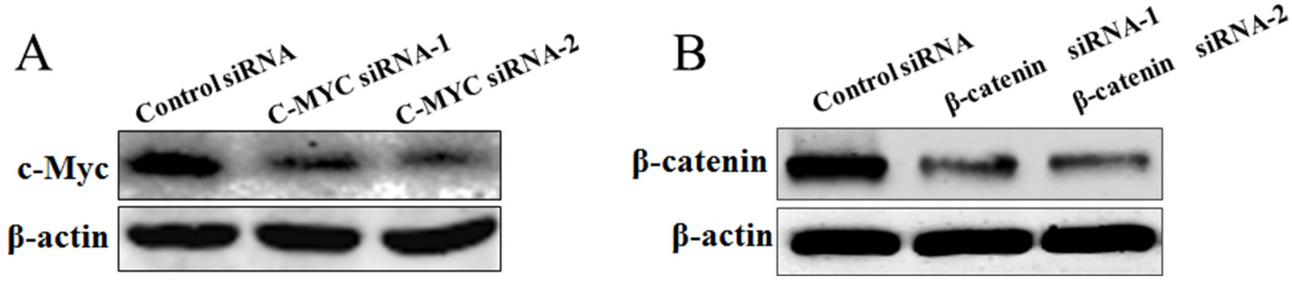

Supplementary Figure S6: The knockdown efficiency of c-Myc and β-catenin expression. **A.** HEK-293T cells were transfected with 60 nM of two different siRNAs targeting c-Myc for 48 h, after which samples were collected for Western blotting with antibodies against c-Myc and β-actin. **B.** HEK-293T cells were transfected with 60 nM of two different siRNAs targeting β-catenin for 48 h, after which samples were collected for Western blotting with antibodies against β-catenin and β-actin.

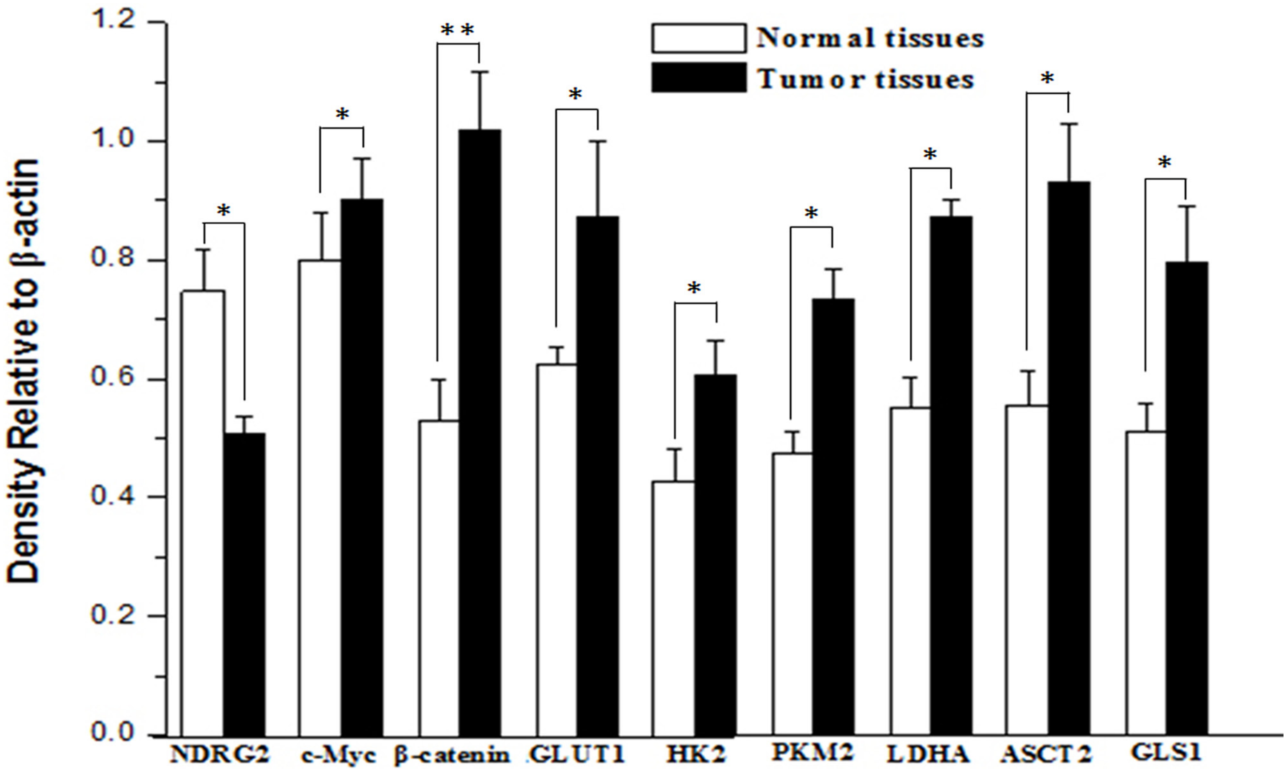

Supplementary Figure S7: Densitometric analysis of the expression of NDRG2 and metabolism- related molecules in clinical colorectal carcinomas. The protein level of NDRG2, c-Myc, β-catenin, glucose transporter GLUT1, glycolytic enzyme HK2, PKM2 and LDHA, glutamine transporter ASCT2, glutaminolytic enzyme GLS1 in the colorectal tumor tissues and adjacent normal tissues were detected by western blot, and β-actin served as an internal control. Densitometric analysis is means ± SEM. of triplicate experiments normalized to β-actin. *,$P < 0.05$; **,$P < 0.01$.

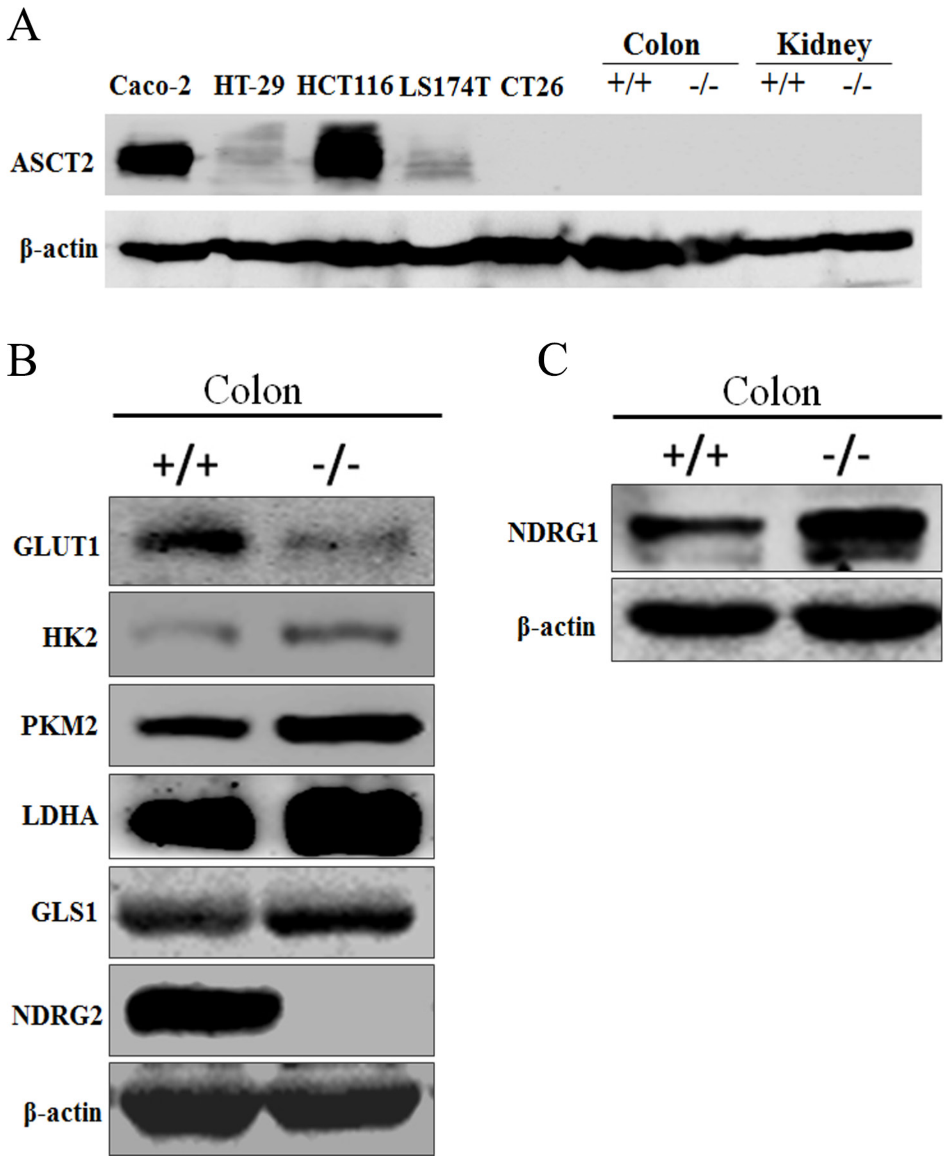

Supplementary Figure S8: Expression analysis of metabolism-related molecules in NDRG2 knock-out mouse. **A.** ASCT2 expression pattern in colon cancer cells and tissues. ASCT2 protein level in human colorectal cancer cell lines Caco-2, HT-29, HCT116 and LS 174T, mouse colorectal cancer cell line CT26, colon tissues and kidney tissues of wide type and NDRG2 knock-out mouse, and β-actin served as an internal control to ensure equal loading. **B.** The protein level of NDRG2, glucose transporter GLUT1, glycolytic enzymes HK2, PKM2 and LDHA, glutaminolytic enzyme GLS1 in NDRG2 knock-out mouse and wild type mouse. **C.** NDRG1 expression in colon tissues of NDRG2 knock-out mouse. NDRG1 protein level in colon tissues of NDRG2 knock-out mouse, and β-actin served as an internal control to ensure equal loading.

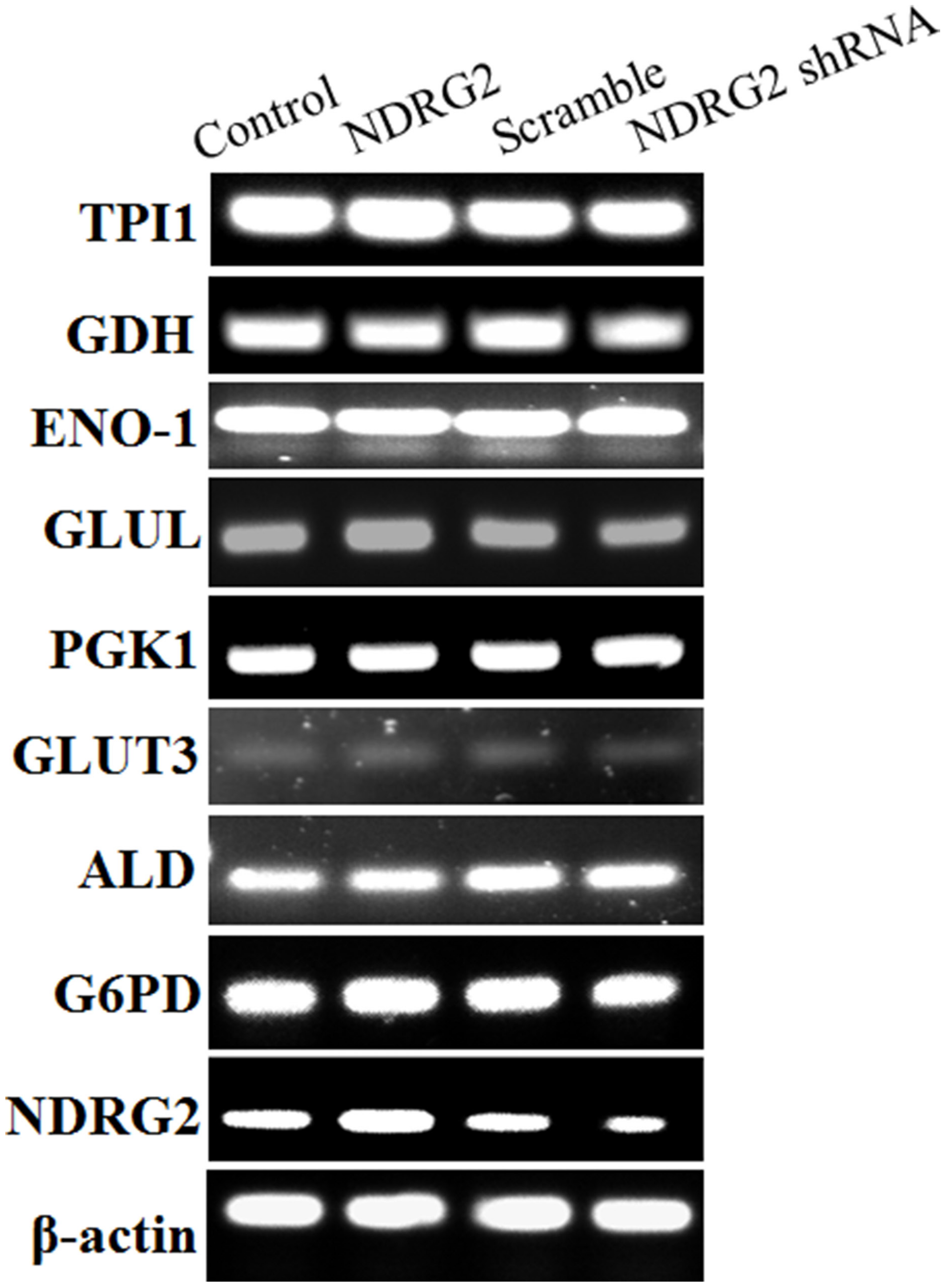

Supplementary Figure S9: The mRNA levels of metabolism-related molecules in NDRG2-overexpressing and NDRG2-knockdown HCT116 cells. The mRNA levels of TPI1, GDH, ENO-1, GLUL, PGK-1, GLUT3, ALD, G6PD, NDRG2 and β-actin in HCT116 cells infected with lentivirus containing NDRG2 or mCherry, NDRG2 shRNA or control shRNA, and β-actin served as an internal control to ensure equal loading.

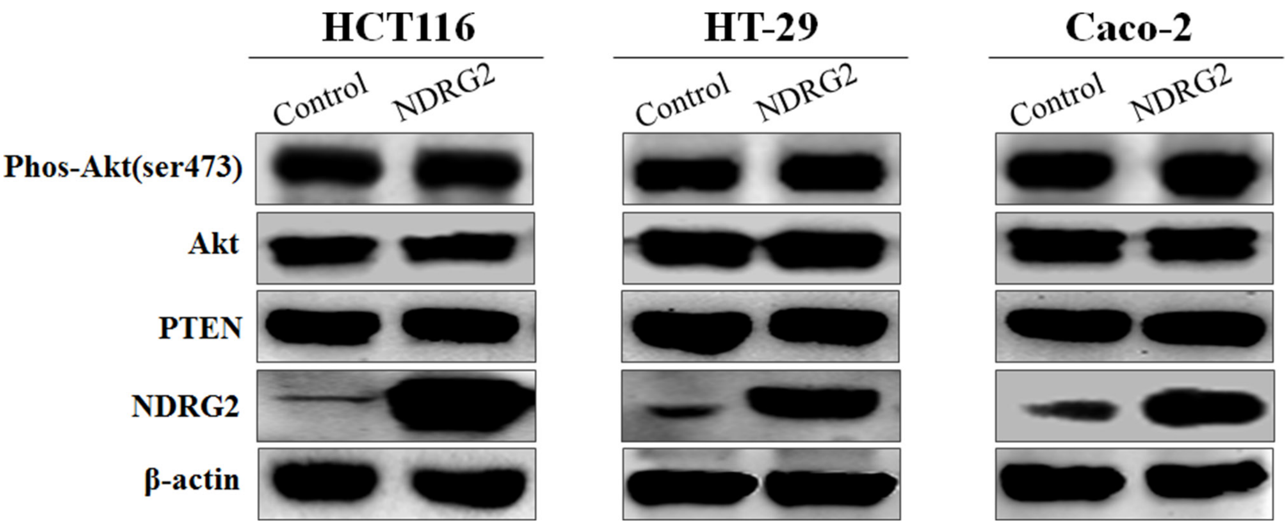

Supplementary Figure S10: The expression and phosphorylation level of PTEN and Akt in NDRG2-overexpressing colorectal cancer cells. The protein levels of PTEN, Akt, phospho-Akt (Ser473) and β-actin in HCT116, HT-29 and Caco-2 cells infected with lentivirus containing NDRG2 or mCherry, and β-actin served as an internal control to ensure equal loading.

Supplementary Table S1: The sequences used for NDRG2 shRNA constructions

shRNA	Sequence
pLKO.1-shNDRG2–1	Forward 5′-CCGGGAGGACATGCAGGAAATCATTCTCGAGAATGATTTCCTGCATGTCCTCTTTTTG-3′
	Reverse 5′-AATTCAAAAAGAGGACATGCAGGAAATCATTCTCGAGAATGATTTCCTGCATGTCCTC-3′
pLKO.1-shNDRG2–2	Forward 5′-AATGATTTCCTGCATGTCCTCACATGCAGGAAATCATTAATTCAAAAAGAGGCTCGAG-3′
	Reverse 5′-TTCCTGCATGTCCTCTTTTTGAGGAAATCATTCTCCCGGGAGGACATGCGAGAATGAT-3′
pLKO.1-shControl	Forward5′-CCGGAAGGTCTTGTCCTCATCAACACTCGAGTGTTGATGAGGACAAGACCTTTTTTTG-3′
	Reverse 5′-AATTCAAAAAAAGGTCTTGTCCTCATCAACACTCGAGTGTTGATGAGGACAAGACCTT-3′

Supplementary Table S2: The siRNA sequences targeting C-MYC and β-catenin

siRNA	Sequence
MYC siRNA1	Sense 5′-CAUCAUCAUCCAGGACUGUAUTT-3′
	Antisense 5′-AUACAGUCCUGGAUGAUGAUGTT-3′
MYC siRNA2	Sense 5′-CGAGCUAAAACGGAGCUUUTT-3′
	Antisense 5′-AAAGCUCCGUUUUAGCUCGTT-3′
β-catenin siRNA1	Sense 5′- AGCUGAUAUUGAUGGACAGTT-3′
	Antisense 5′-CUGUCCAUCAAUAUCAGCUTT-3′
β-catenin siRNA2	Sense 5′- AAGUCCUGUAUGAGUGGGAACTT-3′
	Antisense 5′-GUUCCCACUCAUACAGGACUUTT-3′
Negative Control siRNA	Sense 5′-UUCUCCGAACGUGUCACGUTT-3′
	Antisense 5′-ACGUGACACGUUCGGAGAATT-3′

Supplementary Table S3: The primers used for quantitative real-time RT-PCR

Gene	Forward	Reverse
NDRG2	5′-GAGATATGCTCTTAACCACCCG-3′	5′-GCTGCCCAATCCATCCAA-3′
GLUT1	5′-ACCATTGGCTCCGGTATCG-3′	5′-GCTCGCTCCACCACAAACA-3′
HK2	5′-CCAGTTCATTCACATCATCAG-3′	5′-CTTACACGAGGTCACATAGC-3′
PKM2	5′-GACTGCCTTCATTCAGACCCA-3′	5′-GGGTGGTGAATCAATGTCCAG-3′
LDHA	5′-CTGGGAGTTCACCCATTAAGCT-3′	5′-CAGGCACACTGGAATCTCCAT-3′
ASCT2	5′- CCGCTTCTTCAACTCCTTCAA-3′	5′- ACCCACATCCTCCATCTCCA -3′
GLS1	5′- GCTGTGCTCCATTGAAGTGACT-3′	5′- TTGGGCAGAAACCACCATTAG-3′
C-MYC	5′- ACCACCAGCAGCGACTCTGA-3′	5′-TCCAGCAGAAGGTGATCCAGACT-3′
β-catenin	5′- GCTTTCAGTTGAGCTGACCA-3′	5′- CAAGTCCAAGATCAGCAGTCTC-3′
β-actin	5′-CGCGAGAAGATGACCCAGAT-3′	5′-GTACGGCCAGAGGCGTACAG-3′

Note: Primers shown in Supplementary Table S3 were used to investigate changes in glycolysis and glutaminolysis metabolism.

Supplementary Table S4: The primers used for RT-PCR

Gene	Forward	Reverse
TPI1	5′-GCCCTGGCATGATCAAAGAC-3′	5′- ATCAGCTCATCTGACTCCCCA-3′
GDH	5′-CTCCAGACATGAGCACAGGTGA-3′	5′-CCAGTAGCAGAGATGCGTCCAT-3′
ENO-1	5′-CTGGTGCCGTTGAGAAGGG-3′	5′-GGTTGTGGTAAACCTCTGCTC-3′
GLUL	5′-CTCTTCCAGCCTTCCTTCCT-3′	5′- AGCACTGTGTTGGCGTACAG-3′
PGK-1	5′-CCACTTGCTGTGCCA AATGGA-3′	5′-GAAGGACTTTACCTTCCAG GA-3′
GLUT3	5′-GGAAAGGGCAGGAAGAAGGA-3′	5′- ACAGTCATGAGCGTGGAACAAA-3′
ALD	5′-GTCTGAACTCGCTCACCGC-3′	5′- TGGACTGCAGCCGCTTG-3′
G6PD	5′-TGCAGAACCTCATGGTGCTGAGAT-3′	5′-TCTGGAACCGGGACAACATCGCCT-3′
NDRG2	5′-GAGATATGCTCTTAACCACCCG-3′	5′-GCTGCCCAATCCATCCAA-3′
β-actin	5′-CGCGAGAAGATGACCCAGAT-3′	5′-GTACGGCCAGAGGCGTACAG-3′

Abbreviations: *TPI1*: triosephosphate isomerase 1; *GDH*: glutamate dehydrogenase; *ENO-1*: enolase 1; *GLUL*: glutamate ammonia ligase; *PGK-1*: phosphoglycerate kinase 1; *GLUT3*: glucose transporter 3; *ALD*: acetaldehyde dehydrogenase; *G6PD*: glucose-6-phosphate dehydrogenase.
